# Transcriptome and proteome profiling reveal complementary scavenger and immune features of rat liver sinusoidal endothelial cells and liver macrophages

**DOI:** 10.1186/s12860-020-00331-9

**Published:** 2020-11-27

**Authors:** Sabin Bhandari, Ruomei Li, Jaione Simón-Santamaría, Peter McCourt, Steinar Daae Johansen, Bård Smedsrød, Inigo Martinez-Zubiaurre, Karen Kristine Sørensen

**Affiliations:** 1 Department of Medical Biology, Vascular Biology Research Group, University of Tromsø (UiT) -The Arctic University of Norway, Hansine Hansens veg 18, N-9037 Tromsø, Norway; 2 Faculty of Biosciences and Aquaculture, Nord University, Bodø, Norway; 3 Department of Clinical Medicine, UiT -The Arctic University of Norway, Tromsø, Norway

**Keywords:** Sprague Dawley rat, Sinusoidal endothelial cells, Kupffer cells, Macrophages, Transcriptomics, Proteomics, Immune functions, cell markers, Scavenger receptors

## Abstract

**Background:**

Liver sinusoidal endothelial cells (LSECs) and Kupffer cells (KCs; liver resident macrophages) form the body’s most effective scavenger cell system for the removal of harmful blood-borne substances, ranging from modified self-proteins to pathogens and xenobiotics. Controversies in the literature regarding the LSEC phenotype pose a challenge when determining distinct functionalities of KCs and LSECs. This may be due to overlapping functions of the two cells, insufficient purification and/or identification of the cells, rapid dedifferentiation of LSECs in vitro, or species differences. We therefore characterized and quantitatively compared expressed gene products of freshly isolated, highly pure LSECs (fenestrated SE-1/FcγRIIb2^+^) and KCs (CD11b/c^+^) from Sprague Dawley, Crl:CD (SD), male rats using high throughput mRNA-sequencing and label-free proteomics.

**Results:**

We observed a robust correlation between the proteomes and transcriptomes of the two cell types. Integrative analysis of the global molecular profile demonstrated the immunological aspects of LSECs. The constitutive expression of several immune genes and corresponding proteins of LSECs bore some resemblance with the expression in macrophages. LSECs and KCs both expressed high levels of scavenger receptors (SR) and C-type lectins. Equivalent expression of SR-A1 (Msr1), mannose receptor (Mrc1), SR-B1 (Scarb1), and SR-B3 (Scarb2) suggested functional similarity between the two cell types, while functional distinction between the cells was evidenced by LSEC-specific expression of the SRs stabilin-1 (Stab1) and stabilin-2 (Stab2), and the C-type lectins LSECtin (Clec4g) and DC-SIGNR (Clec4m). Many immune regulatory factors were differentially expressed in LSECs and KCs, with one cell predominantly expressing a specific cytokine/chemokine and the other cell the cognate receptor, illustrating the complex cytokine milieu of the sinusoids. Both cells expressed genes and proteins involved in antigen processing and presentation, and lymphocyte co-stimulation.

**Conclusions:**

Our findings support complementary and partly overlapping scavenging and immune functions of LSECs and KCs. This highlights the importance of including LSECs in studies of liver immunity, and liver clearance and toxicity of large molecule drugs and nano-formulations.

**Supplementary Information:**

The online version contains supplementary material available at 10.1186/s12860-020-00331-9.

## Background

The liver has a central role in host defense [1, 2]. Its extensive capillary network, the sinusoids, houses the body’s most effective scavenger cell system comprising the Kupffer cells (KCs; the body’s largest reservoir of resident macrophages [3]), and liver sinusoidal endothelial cells (LSECs). For decades KCs, facing the sinusoidal lumen, were believed to be the only liver cell responsible for the clearance of blood-borne material [4, 5]. This view was challenged by a series of studies throughout the 1980s and 1990s showing that a number of physiological macromolecules and colloids were cleared chiefly by LSECs, but only to a minor extent by KCs [6–15]. Today it is accepted that LSECs and KCs together make up the hepatic “dual cell principle of waste clearance”, with LSECs being geared to effective clathrin-mediated endocytosis of nanoparticles (< 200 nm), colloids, and macromolecules, and KCs taking up larger material [5]. The discovery that these cells share the task of blood clearance in this way suggested that LSECs are a highly specialized endothelium with characteristics in common with KCs, not only functionally, but at the molecular level as well. The present study was undertaken to study the similarities and differences of the two cells, by comparing their transcriptomes and proteomes.

The liver receives approximately 25% of cardiac output, exposing the sinusoidal cells to large volumes of blood, thus placing these cells in a unique position to monitor blood content. Approximately 80% of the organ blood supply drains the gut and contains (in addition to nutrients) toxins, bacterial components, viruses, and various waste products that are efficiently removed from blood by uptake in LSECs and KCs [5, 15], thus preventing deposition and deleterious effects of such components elsewhere. LSECs show an extraordinarily high capacity for uptake of soluble macromolecules and nanoparticles, including virus [10, 11, 15–23]. For this purpose, LSECs express several high affinity endocytosis receptors, some of which are pattern recognition receptors. These include the scavenger receptors (SRs) stabilin-1 and stabilin-2 [24, 25], the macrophage mannose receptor (CD206) [17], and the endocytic Fc-gamma receptor IIb2 (FcγRIIb2, CD32b) [26]. In addition, LSECs express several Toll-like receptors (TLRs) [27–29], and in mice, the cells are reported to possess adaptive immune functions, including cross-presentation of endocytosed antigens to naïve CD8^+^ T-cells contributing to the generation of memory T-cells important for liver immune tolerance [1, 27, 30–32]. In contrast to KCs, LSECs are normally not phagocytic but can take up 1 μm particles if KCs are depleted [33].

Due to the overlapping functions of LSECs and KCs as scavenger cells [1, 2, 5], the large endothelial cell diversity between different vascular beds [34, 35], and the lack of standardized methods for LSEC isolation and identification between different research groups [36, 37], LSECs have been described as a cell of controversial and confusing identity [37]. For instance, the pan-leukocyte marker CD45 is often used as a negative selection criterion for isolation of mouse and human LSECs by immune based methods but is reported to be expressed in rat LSECs [36, 38]. Furthermore, LSECs rapidly dedifferentiate in culture [39, 40], which poses a problem for long-term co-cultures with e.g. lymphocytes in immune assays. This highlights the importance of using early primary cells when exploring cell functions and molecular expression patterns, and mapping LSEC and KC gene and protein expression in different species used in biomedical research.

In order to resolve some of the discrepancies in the literature regarding LSEC and KC markers and molecular phenotypes, we directly compared the transcriptome and proteome of freshly isolated rat LSECs and KCs. Studies comparing the gene/protein expression of LSECs and KCs are rare. To the best of our knowledge only two studies, both done in C57Bl/6 mice, have compared the proteome of liver resident cell populations [41, 42], but without discussing LSEC scavenger or immune functions. Our study represents the first comprehensive multiomics profiling and comparison of rat KCs and LSECs. Based on our findings we conclude that LSECs differ from other types of endothelial cells due to their distinct immunological features.

## Results

### Isolation of LSECs and KCs using SE-1 and CD11b/c yields highly pure cell preparations

**Fig. 1:**
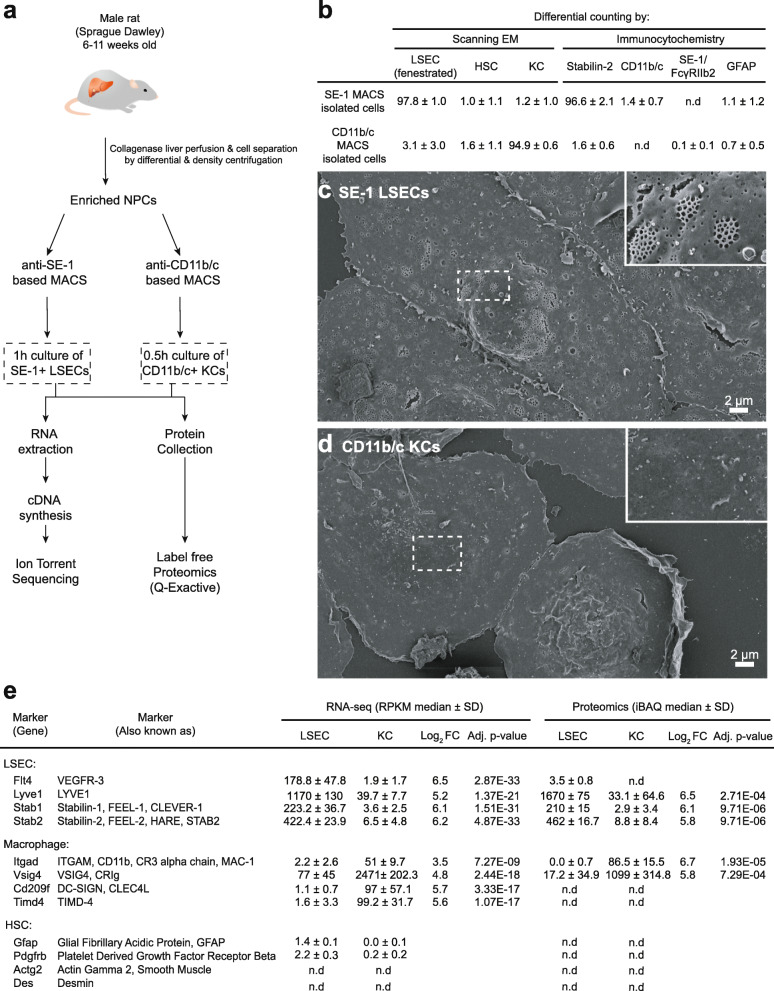
Overview of experimental workflows and cell purity tests. **a**. Schematic overview of the high-throughput transcriptomics and label-free proteomics workflows. **b**. Purity of SE-1-MACS-isolated liver sinusoidal endothelial cells (LSECs) and CD11b/c-MACS-isolated Kupffer cells (KCs). Cell isolates were analyzed by scanning electron microscopy (EM) (LSECs: *n* = 6, including all cell isolates for proteomics and RNA sequencing; KCs: *n* = 4, including all isolates for proteomics), and immune cytochemistry (KC: *n* = 4, LSEC: *n* = 3, including all cell isolates for proteomics). Results are presented as % of total cell count (mean ± standard deviation). Antibodies (Table 1) targeted either stabilin-2 (LSEC marker), SE-1/FcγRIIb2 (LSEC marker), CD11b/c (KC marker), or glial fibrillar acidic protein (GFAP, stellate cell marker). N.d., not determined. **c-d**. Scanning electron micrographs showing the typical morphology of MACS-isolated cells. Insert in c shows LSEC fenestrations (hallmark of LSECs), which were absent in KCs (d). **e**. Expression level of marker genes for LSECs, KCs, and hepatic stellate cells (HSC) in the KC and LSEC transcriptomes and proteomes. Expression values are given as RPKM (RNA-seq), and iBAQ (label-free proteomics), as described in Methods

An overview of the transcriptomics and proteomics experiments and purity tests of cells used in the experiments is given in Fig. 1. LSECs and KCs were purified by magnetic-activated cell separation (MACS) of non-parenchymal liver cell (NPC) suspensions generated from collagenase perfused rat liver, then plated for 0.5 h (KCs) or 1 h (LSECs) and washed with medium before RNA and protein extraction (Fig. 1a). For LSEC, we used the SE-1 monoclonal antibody [43, 44] (Table 1), which targets FcγRIIb2 [45] and has been previously tested for MACS-based purification of rat LSECs [43]. The isolated cells were > 97% LSECs (i.e. fenestrated endothelial cells), as examined by scanning electron microscopy (SEM), and 96.6% were stabilin-2 positive by immune staining (Fig. 1b-d). The few contaminating cells were KCs and stellate cells. A monoclonal antibody to CD11b/c (Table 1), targeting complement receptor 3 (CR3) was used to purify KCs. This yielded 94.9% KCs - contaminating cells were 3.1% LSECs and 1.6% stellate cells (Fig. 1b).

**Table 1:** Antibodies used in the study

Antibody (clone)	Target	Company/Reference	Catalog #	Working concentration
***Flow cytometry antibodies and isotype controls***				
CD45-PE (OX-1)	CD45, PTPRC	Novus Biologicals	NB100–64895PE	0.85 μg/ million cells
PE Mouse IgG1	IgG1 κ isotype control	BD Pharmingen	555749	0.2 μg/ million cells
HSEC^a^ antibody (SE-1) -AF488	CD32b, FcγRIIb2	Novus Biologicals	NB110–68095AF488	1 μg/ million cells
Mouse IgG2a AF488 (MG2a-53)	IgG2a κ isotype control	Novus Biologicals	NB600-986AF488	0.65 μg/ million cells
CD31-eFluor 660 (TLD-3A12)	CD31, PECAM-1	eBioscience	50–0310-82	0.2 μg/ million cells
Mouse IgG1k- eFluor 660 (P3.6.2.8.1)	IgG1 κ isotype control	eBioscience	50–4714	0.2 μg/ million cells
***Immune staining of cells and tissues***				
HSEC^a^ antibody (SE-1)	CD32b, FcγRIIb2	Novus Biologicals	NB110–68095	10 μg/ml
CD11b/c Biotin (OX-42)	CD11b/c, CR3	Cedarlane	CL042B	2 μg/ml
CD163 (ED2)	CD163	AbD Serotec	MCA342GA	10 μg/ml
CD68 (ED-1)	CD68 antigen, macrosialin	Abcam	ab31630	20 μg/ml
GFAP	Glial fibrillary acidic protein	Dako	Z0334	15 μg/ml
Human MMR/CD206	CD206, macrophage mannose receptor	R&D Systems	AF2534	2 μg/ml
SR-A1/MSR	Macrophage scavenger receptor A1	Novus Biologicals	NBP1–00092	12 μg/ml
SR-B1	Scavenger receptor B1	Novus Biologicals	NB400–104	10 μg/ml
Rabbit anti-rat HA/SR serum^b^	Stabilin-2, STAB2	(24)		1:200
CD45 (OX-1)	CD45, PTPRC	Novus Biologicals	NB100–64895	10 μg/ml
CD31 (TLD-3A12)	CD31, PECAM-1	Invitrogen	MA1–81051	10 μg/ml
***Magnetic-activated cell sorting***				
HSEC^a^ antibody (SE-1)	CD32b, FcγRIIb2	Novus Biologicals	NB110–68095	0.2 μg/million NPCs
CD11b/c Biotin (OX-42)	CD11b/c, CR3	Cedarlane	CL042B	0.1 μg/million NPCs
Anti-Mouse IgG2a + b MicroBeads	IgG2a + b	Miltenyi	130–047-201	2 μl/million NPCs

^a^HSEC, hepatic sinusoidal endothelial cell

^b^Stabilin-2 was named the hyaluronan-scavenger receptor (HA/SR) in reference [24]

Secondary antibodies used for immune labeling of cells and tissues were all species-matched AlexaFluor antibodies from Invitrogen (ThermFischer)

Quantitative expression of marker genes used for cross validation of the transcriptomics and proteomics data are listed in Fig. 1e. Consistent with SEM and immunocytochemistry analysis of MACS-isolated cells, expression of macrophage and stellate cell markers were low in the LSEC transcriptomes and proteomes, whereas expression of LSEC and stellate cell markers were low in the KC transcriptomes and proteomes.

**Fig. 2:**
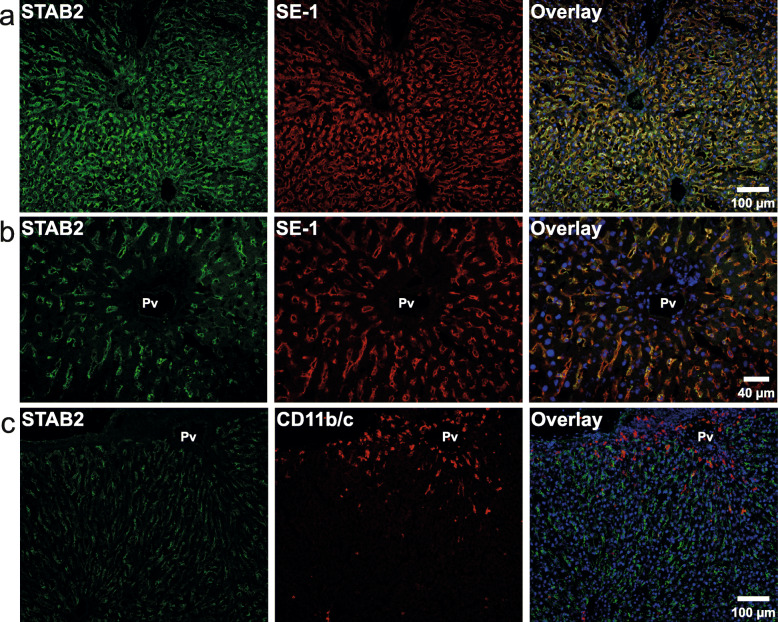
Immune histochemistry of acetone-fixed frozen rat liver sections. The sections were double immune-labeled with primary antibodies against **a-b**) stabilin-2 (STAB2; green fluorescence), and FcγRIIb2 (SE-1; red fluorescence), or against **c**) stabilin-2 (STAB2; green fluorescence), and CD11b/c (red fluorescence). Antibodies are listed in Table 1. Pv, portal vein. Nuclei were stained with DAPI (blue fluorescence in overlay images)

To check the hepatic intralobular distribution of cells expressing SE-1 (i.e. FcγRIIb2), and CD11b/c, frozen rat liver sections were stained with the same antibodies used for MACS-isolation of cells (Fig. 2). The SE-1 antibody showed a strict sinusoidal staining pattern, colocalizing with the LSEC marker stabilin-2 [24, 46, 47] in all sinusoids (Fig. 2a, b). Most CD11b/c positive cells were located in the periportal region and showed a different staining pattern than stabilin-2 (Fig. 2c).

### Global information generated from omics data profiling

**Fig. 3:**
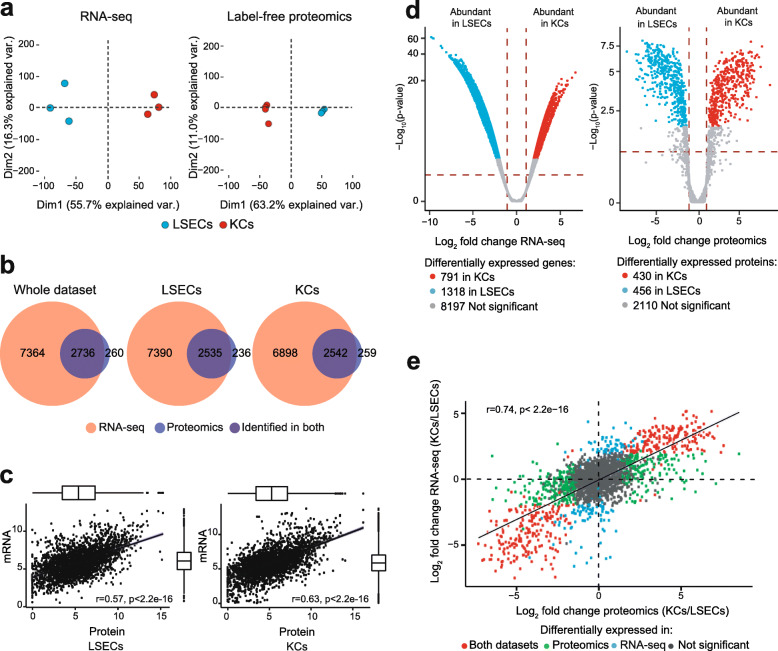
Global characterization and comparison of the LSEC and KC transcriptome and proteome datasets. **a**. Principal component analysis (PCA) plot displaying distinct clusters of the LSEC and KC samples in transcriptome and proteome datasets created by high-throughput mRNA sequencing (RNA-seq), and label-free proteomics (LFP). PCA plots are generated from normalized log_2_ expression values (RPKM for RNA-seq, and iBAQ for LFP). **b**. Venn diagrams illustrating the number of gene products identified in the respective experiments (RNA-seq, LFP), and their overlap. **c**. Scatter plots illustrating the global correlation between the RNA-seq data and the LFP data. Results for LSECs and KCs are shown separately. **d**. Volcano-plot illustrating differently expressed genes. Blue dots: significantly higher expression in LSECs; red dots: significantly higher expression in KCs; gray dots: not significantly different between LSECs and KCs. Significance level: FDR ≤ 0.05 and |log_2_ fold change| ≥ 1. **e**. Scatter plot showing correlation of KC vs. LSEC log_2_ fold change values for all features expressed in both the transcriptome and proteome datasets. The r value of 0.74 indicates that approximately two thirds of the gene products are consistently significantly differentially expressed between LSECs and KCs with respect to mRNA and protein expression (FDR ≤ 0.05 and |log_2_ fold change| ≥ 1)

In the RNA-seq experiment 10,306 genome features were deemed expressed and included in the subsequent analyses, while in the label-free proteomics experiment 2996 non-redundant protein IDs were deemed expressed and included in the further analyses. Principal component analysis (Fig. 3a) segregated the LSEC and KC samples into disparate clusters coherent with the distinct biology of the cells.

Figure 3b illustrates the total number of gene products identified with the respective techniques, and their overlap, in the LSEC and KC groups. The proteome covered 26–27% of the transcriptome. Notably, most proteins (90.8–91.5%) identified in the proteome had valid corresponding mRNA in the transcriptome. To evaluate the coherence between the transcriptome and proteome, we calculated the global Pearson correlation coefficient r using the expression data between the omics datasets for each cell type. The global correlation r value was 0.57 for LSECs, and 0.63 for KCs (Fig. 3c) which are in the upper end of the previously reported range of 0.4–0.6 [41, 48] supporting the reliability of the data.

Differentially expressed gene products are key to understanding phenotypic and functional variation between cell types. The results of the differential expression analyses of the RNA-seq data, and the proteomics data are summarized in Fig. 3d. We identified 2109 gene products in the transcriptome (20.5%) as significantly differentially expressed (with cutoff of FDR (false discovery rate) ≤ 0.05 and |log_2_ fold change| ≥ 1) in LSECs and KCs. Similarly, in the proteome, 886 proteins (~ 30%) were significantly differently expressed in the two cells (with cutoff of FDR ≤ 0.05 and |log_2_ fold change| ≥1). Despite differences in percentage of differentially expressed gene products in the RNA-seq and proteomics experiments, the log_2_ fold changes for the unique gene products identified in both datasets showed high correlation (r = 0.74 [95% CI: 0.72–0.75]) (Fig. 3e), suggesting good congruence between the two techniques.

### LSECs and KCs show enrichment of terms reflecting their ontogeny

**Fig. 4:**
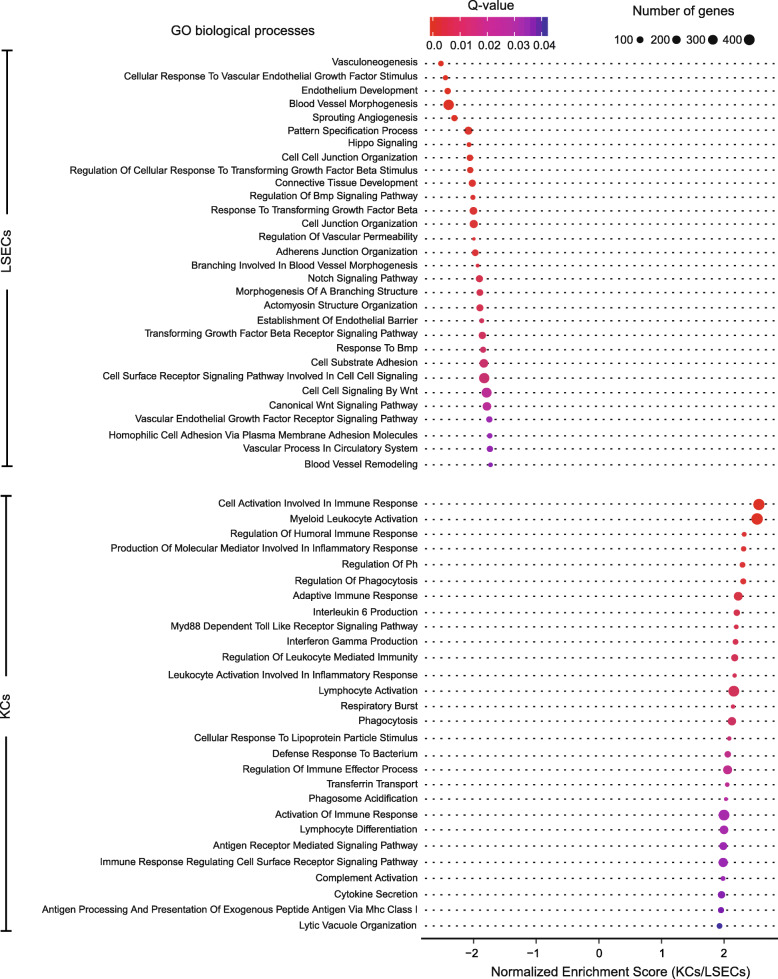
Dot plot showing selected enriched terms in KC and LSEC transcriptomes, belonging to GO biological processes in the Molecular Signatures Database (MSigDB) [53]. The Normalized Enrichment Score reflects the degree of overrepresentation of the genes in a gene set across the entire ranked list of genes after adjusting for differences in gene set size, and correlation between the gene sets and the RNA-seq expression data. Dot size represents the number of genes assigned to the specific process, and dot colour represents the associated FDR q-value generated from the GSEA analysis

We used ranked gene lists based on expression level from the RNA-seq experiment as input for gene set enrichment analysis (GSEA) [49, 50] to identify the intrinsic functional characteristics of LSECs and KCs. GSEA showed enrichment of 268 biological processes in LSECs and 121 biological processes in KCs with FDR q-value ≤0.05 corresponding to Gene Ontology (GO) terms [51, 52] in the Molecular Signatures Database [49, 53] that concur with the generic role of these cells (Additional file 1; Fig. 4). Like other endothelial cells, LSECs are involved in development, morphogenesis, patterning and maintenance of blood vessels, and displayed enrichment of gene sets associated with response to vascular endothelial growth factor and regulation of WNT, BMP, and TGFβ signalling pathways. KCs, being macrophages, displayed enrichment of terms related to adaptive and innate immune responses.

Expression of genes associated with endocytic function, cytoskeleton organization, and positive regulators of endocytosis, such as 1-phosphatidylinositol-4-phosphate 5-kinase (Pip5klc), phospholipase D1/2 (Pld2), integrin subunit beta1 (Itgb1), GTPase Hras, clathrin adaptor protein (Dab2), caveolin1 (Cav1), and E3 ligase NEDD4 (Nedd4) were higher in LSECs than in KCs (Additional file 2). Moreover, LSECs showed higher expression of transport-related proteins such as EH domain-containing protein 3 (Ehd3), which is suggested to be involved in transport of stabilin-1-positive vesicles [39], adaptor-related protein complex 1 beta 1 subunit (Ap1b1), and sorting nexin (Snx) 8 and 33, which are associated with vesicular transport (Additional file 2). Interestingly, RNA-seq of LSECs revealed high expression of genes coding for connective tissue components such as Sparc, Col4a1, Col4a2, Egfl7, and Mfge8, indicating a significant role of these cells in extracellular matrix maintenance and remodeling of liver (Additional file 2). Transcription factor Gata4, which is essential for LSEC differentiation [39, 47] was specifically expressed in the LSEC transcriptome (Additional file 2).

### Most gene products involved in KC immune functions are also expressed in LSECs

**Fig. 5:**
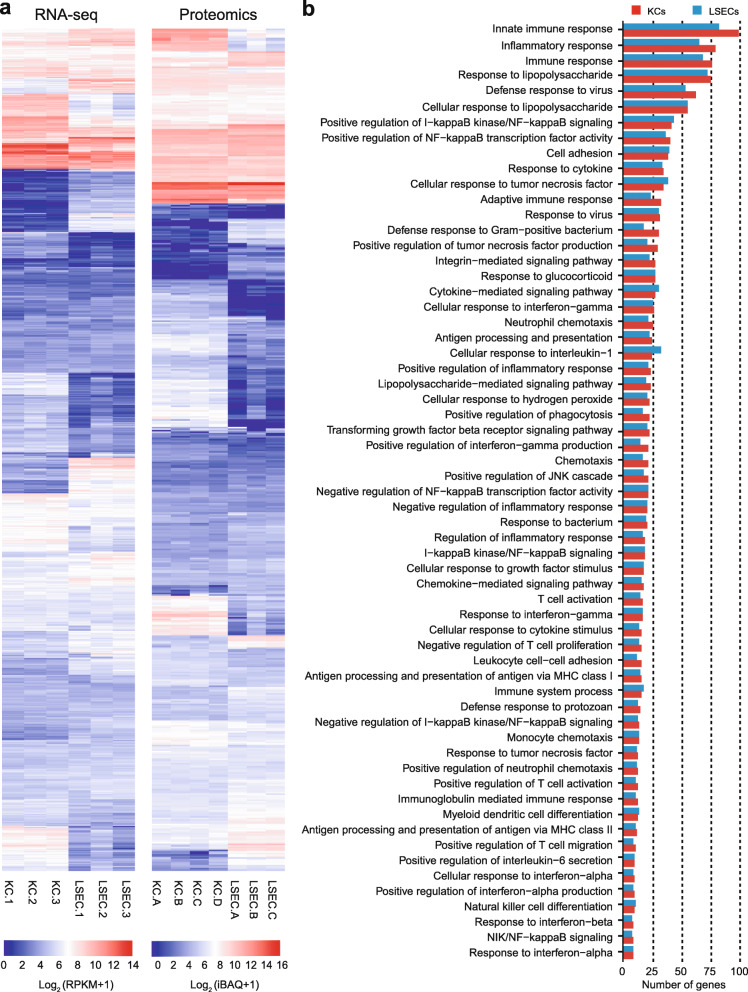
Expression of immune genes in rat LSECs and KCs. **a**. Unscaled heatmaps of normalized log_2_ expression values (log_2_ (RPKM+ 1), and log_2_ (iBAQ+ 1)) for all gene products associated with the term immune system processes (GO:0002376) in the KC and LSEC transcriptome and proteome. **b**. The figure shows significantly enriched GO terms (FDR ≤ 0.05) associated with immune functions, and the density of corresponding genes with expression ≥10 RPKM in the LSEC and KC transcriptomes

Genes associated with the term immune system processes (GO:0002376) include 2645 annotated objects in the rat genome database (December 13, 2019). Of these, we found 1466 expressed genes in the RNA-seq data, and 554 expressed genes in the label-free proteomics experiments that were associated with the term (Fig. 5a; Additional file 3). Both cells expressed numerous immune genes - the majority of which were expressed at low density but more abundant in KCs compared to LSECs. To ascertain the immunological role of expressed genes we performed functional enrichment analysis (DAVID 6.8 [54, 55]) of genes with expression values ≥10 RPKM (reads per kilobase of exon model per million mapped reads [56]) separately in the LSEC and KC RNA-seq datasets. The threshold 10 RPKM was set to increase the confidence of the results. The immune terms that were significantly enriched (FDR ≤ 0.05) in KC and LSEC transcriptomes were similar, and each term contained almost similar number of expressed genes in the two cells (Fig. 5b).

### Both cell types show high expression of scavenger receptors and immune lectins

**Fig. 6:**
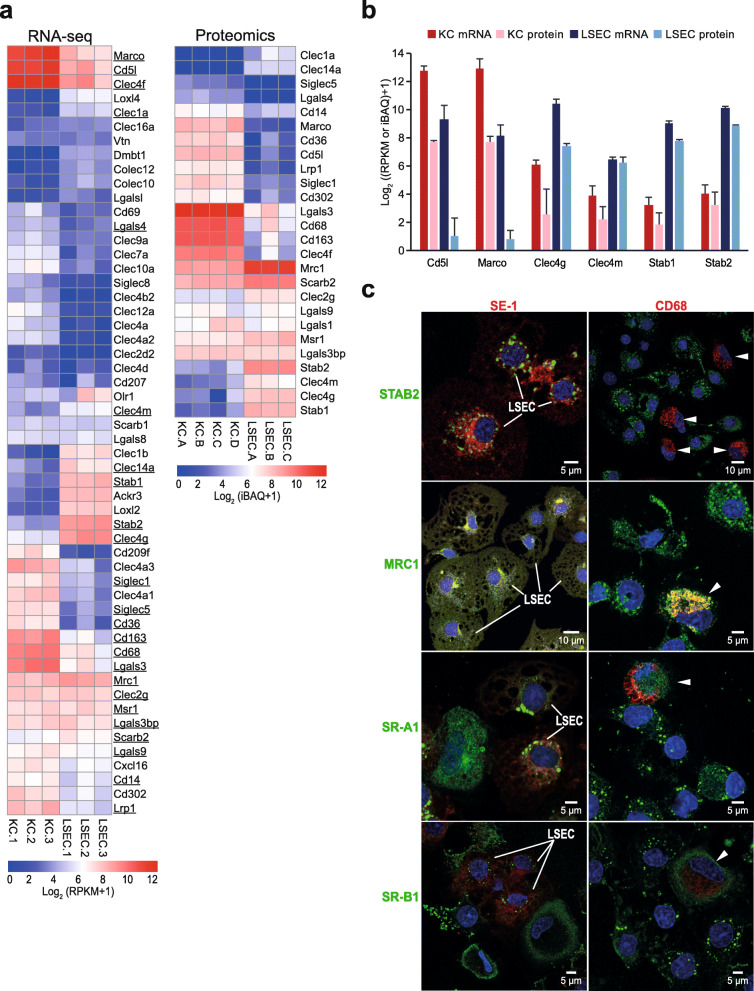
Expression of scavenger receptors and immune lectins in rat LSECs and KCs. **a**. Unscaled heatmaps of normalized log_2_ expression values (log_2_ (RPKM+ 1), and log_2_ (iBAQ+ 1)) for scavenger receptors (SR) and C-type lectins in the KC and LSEC transcriptomes and proteomes. Underlined: Genes expressed in the transcriptome that were also present in the proteome. **b**. Absolute abundance of selected SR gene products in the KC and LSEC transcriptomes and proteomes. The bar height reflects good correlation between the transcriptome and proteome data for gene products of Clec4g, Clec4m, Stab1, and Stab2 in both cell types. The abundance of gene products of Marco and Cd5l were well correlated between the KC transcriptome and proteome, while LSECs showed high abundance of these gene products only at mRNA level. **c**. Immune labeling of non-parenchymal liver cell (NPC) cultures for selected SRs and C-type lectins. NPCs from the 25–45% interface on the Percoll gradient were incubated for 1 h, then fixed 15 min in 4% paraformaldehyde, and double immune-labeled with antibodies to FcγRIIb2 (SE-1; red fluorescence; left column), or CD68 (red fluorescence; right column), and to either stabilin-2 (STAB2; green), mannose receptor (MRC1; green), SR-A1 (green), or SR-B1 (green). Overlap of green and red fluorescence is seen as yellow staining in the overlay images. Antibodies are listed in Table 1. Cell nuclei were stained with DAPI (blue). Arrow heads point to CD68 positive KCs. Antibodies to stabilin-2 and FcγRIIb2 (SE-1) specifically labeled LSECs and the CD68-antibody specifically labeled KCs, whereas positive labeling for the mannose receptor, SR-A1, and SR-B1 was observed in both LSECs and KCs

LSECs and KCs express a variety of SRs, C-type lectins, and TLRs [16, 17, 27–29]. We found that both cells expressed many SRs and immune lectin gene products at high densities, of which some were cell type specific (Fig. 6a; Additional file 4), providing the capacity of rapid sensing and clearance of various danger molecules. Among these were the macrophage mannose receptor (Mrc1) and macrophage SR-A1 (Msr1) which were abundantly expressed both in the LSEC and KC transcriptomes and proteomes (Fig. 6a) and confirmed by immune cytochemistry (Fig. 6c). The high-density lipoprotein receptor SR-B1 (Scarb1) was also equally expressed in the rat LSEC and KC transcriptomes, but at low density, and were not identified in the cell proteomes. However, immune labelling experiments validated SR-B1 protein expression in both LSECs and KCs (Fig. 6c), in accordance with [57]. Of note, CD36, a reliable LSEC marker in human liver [58] was evidently expressed in rat KCs but was very low in rat LSECs (Fig. 6a). Same receptor was previously reported to be absent from Sprague Dawley rat LSECs in western blot and immune fluorescence experiments [59].

Stabilin-1 (Stab1) and stabilin-2 (Stab2) were expressed at much higher densities in the LSECs than in KCs (Fig. 6a-b). Immune labeling of NPCs (Fig. 6c) and frozen rat liver sections (Fig. 2) for stabilin-2 confirmed LSEC specific expression and a typical LSEC distribution pattern in all hepatic zones of this protein, in accordance with [60] supporting the use of stabilin-2 as a specific pan-LSEC marker. Furthermore, rat LSECs showed high mRNA and protein expression of Clec4g (LSECtin) and Clec4m (DC-SIGNR) (Fig. 6a, b), as was also reported in a study of human LSECs [61], where Clec4g was used as a specific LSEC marker in liver single cell transcriptome studies [62].

Some of the receptors reported in the literature to discriminate KCs from other liver cells, were also expressed in the LSEC transcriptome. These included Marco, Cd5l, Clec4f, Cd163, lgals3, and Cd68 (Fig. 6a, b). However, their transcript level in KCs were significantly higher compared to LSECs, and their abundance in the LSEC proteome was low. Immune labeling of NPCs for CD163 (not shown) and CD68 showed staining of KCs only (Fig. 6c) and labeling of rat liver sections for CD68 together with the LSEC marker stabilin-2 showed a staining pattern of CD68 that is typical for KCs (Additional file 5), supporting the proteomic results.

Several TLRs were detected in LSECs and KCs transcriptomes (Additional file 3). The abundance of Tlr4, 5, 6, 7, 8, 10, 11, and 12 mRNA was significantly higher (FDR ≤ 0.05) in KCs, whereas Tlr2, 3, and 13 were not significantly different. The only TLR identified by proteomics at steady state was TLR3 which was identified in both cells.

### Immune regulatory factors expressed by LSECs and KCs

**Fig. 7:**
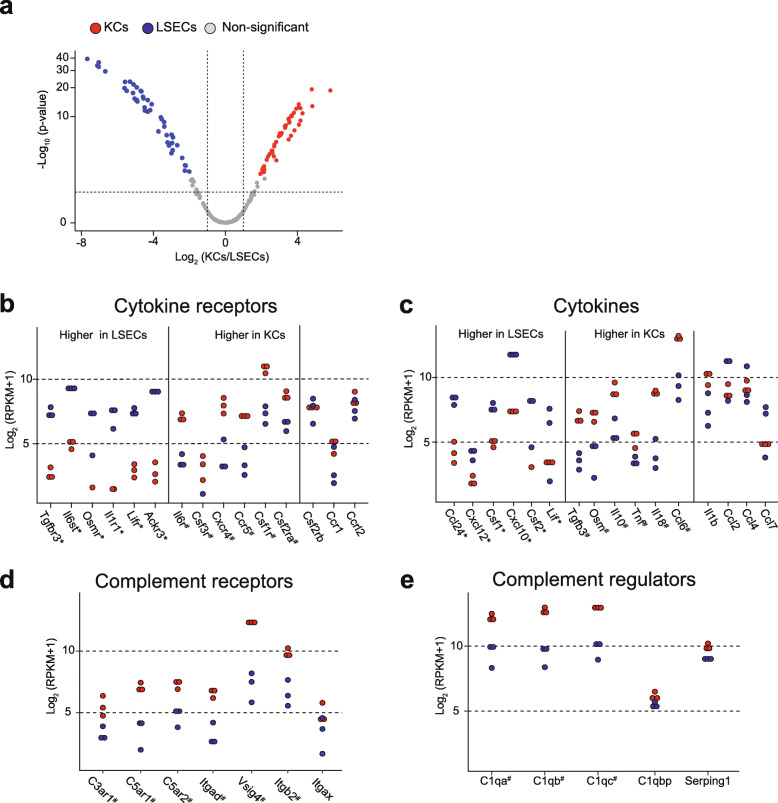
Expression of immune regulatory factor genes in the rat LSEC and KC transcriptomes. The genes were associated with the terms cytokine receptor activity (GO:0004896), cytokine receptor binding (GO:0005126), complement receptor activity (GO:0004875), and complement activation (GO:0006956). **a**. Volcano-plot illustrating differently expressed genes. Blue dots: significantly higher expression in LSECs; red dots: significantly higher expression in KCs; gray dots: not significantly different between LSECs and KCs. Significance level: FDR ≤ 0.05 and |log_2_ fold change| ≥ 1. **b-e**. Expression of selected genes in LSECs and KCs transcriptome associated with cytokine receptor activity (**b**), cytokine activity (**c**), complement receptor activity (**d**), and regulators of complement activation (**e**). Each blue dot represents abundance, corresponding to log_2_ expression values (RPKM+ 1), in an LSEC sample and each red dot represents abundance in a KC sample. *Significantly higher in LSECs, ^#^significantly higher in KCs. Significance level: FDR ≤ 0.05 and |log_2_ fold change| ≥ 1

When reviewing genes annotated with cytokine receptor binding (GO:0005126), cytokine receptor activity (GO:0004896), complement activation (GO:0006956), and complement receptor activity (GO:0004875), we identified 209 genes in the transcriptome (out of 551 objects associated under the terms), and 54 proteins in the proteome (Additional file 6). Low protein identification may be due to the fact that these genes are normally expressed at low levels in non-stimulated cells from healthy animals (as analyzed in this study), and many gene products associated with the terms represent secreted proteins, mostly found extracellularly. Thus, the bulk of gene products affiliated with the terms were only detected in the transcriptome, and at low level. Many were also differently expressed in the LSEC and KC transcriptomes (Fig. 7a).

Figure 7b-c reflects the complex cytokine milieu of the sinusoids. LSECs showed significantly higher expression of the cytokine receptors Tgfbr3, Il6st, Osmr, Il1r1 and Lifr (Fig. 7b) enabling them to sense and respond to the cytokines Tgfb3, Osm, Il1b and Lif in paracrine and autocrine manners. Tgfb3, Osm, and Il18 were more abundantly expressed by KCs (Fig. 7c). LSECs also expressed high levels of Ackr3 (Fig. 7b) which is involved in scavenging and degradation of chemokines, thus regulating their levels in the hepatic sinusoids. KCs showed significantly higher expression of the cytokine receptors Il6r and Csf3r, and chemokine receptor Cxcr4 (Fig. 7b), which allow KCs to respond to Ccl24, Cxcl12, Ccl2, Ccl6, and Ccl7 in an autocrine or paracrine manner (Fig. 7c).

The expression of colony stimulating factor receptors Csf1r, Csf2ra and Csf3r were also higher in KCs (Fig. 7b). Of these, Csf1r and Csf2ra were detected by proteomics, being significantly higher in KCs (Additional file 6). Interaction of colony stimulating factor receptors with their ligands, e.g. Csf1 and Csf2 which were abundantly expressed in LSECs (Fig. 7c), affects KC maturation [63], underlining the importance of LSECs for proper KC function.

The complement system is an important part of the innate immune system. Hepatocytes are major producers of complement proteins, whereas NPCs regulate complement activation [42]. Gene products representing complement receptors (Fig. 7d), and triggers of complement activation (C1qa, C1qb, C1qc; Fig. 7e) were significantly more abundant in the KC transcriptome and proteome datasets, whereas the expression of the C1 inhibitors C1qbp and Serping1 was similar in the two cells (detected only in the transcriptome; Fig. 7e).

### LSECs express the machinery needed for antigen presentation and lymphocyte activation

**Fig. 8:**
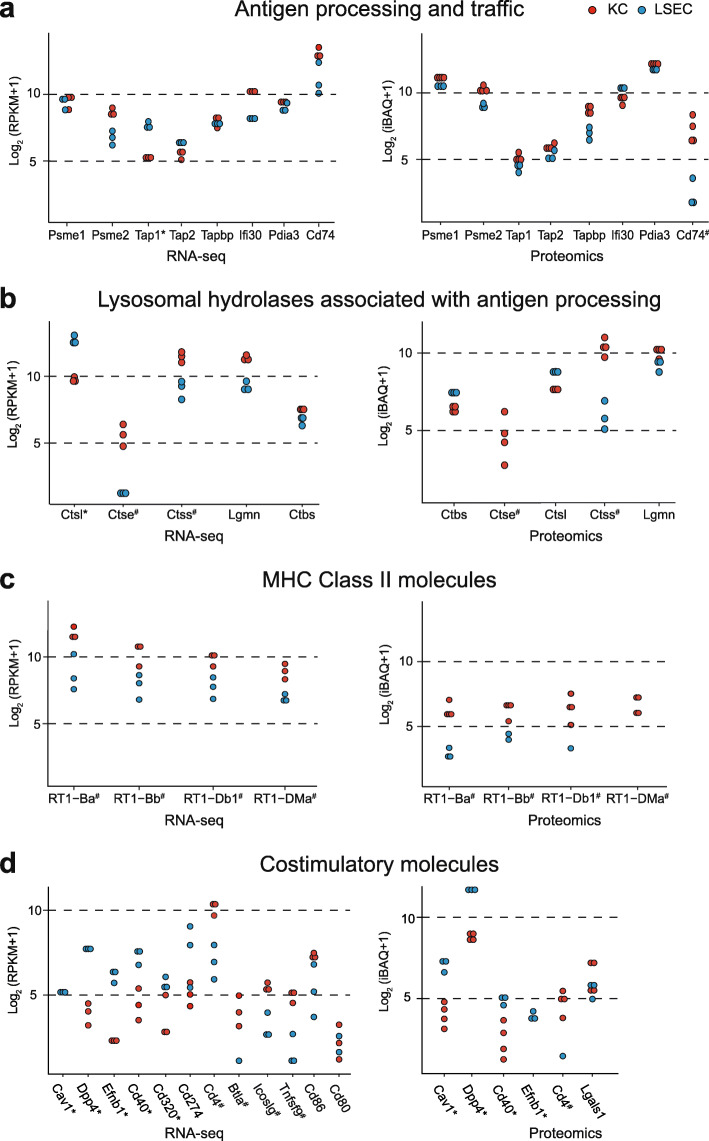
Expression of selected gene products associated with antigen processing and presentation (GO:0019882), and lymphocyte co-stimulation (GO:0031294) in the rat LSECs and KCs transcriptome and proteome. Each blue dot represents abundance of gene products in an LSEC sample and each red dot represents abundance in a KC sample. The dot plots on the left illustrate the abundance of mRNA (log_2_ (RPKM+ 1)) in the transcriptome, and the right plots illustrate the corresponding gene expression value in the proteome (log_2_ (iBAQ+ 1)). *Significantly higher in LSECs, ^#^significantly higher in KCs. Significance level: FDR ≤ 0.05 and |log_2_ fold change| ≥ 1. **a**. Gene products involved in antigen processing (immune proteases: Psme1, Psme2), transport of processed peptide into the endosome for loading into MHC molecules (Tap1, Tap2, Tapbp), accessory proteins in loading and sorting of MHC molecules to endolysosome (Ifi30, Pdia3), and the invariant chain (Cd74). **b**. Lysosomal hydrolases annotated to be associated with MHC class II antigen processing. **c**. MHC class II gene products. **d**. Co-stimulatory factors involved in lymphocyte activation

A series of studies in mouse models suggest that LSEC cross-presentation of exogenous soluble antigens to naïve T cells is central to maintaining liver immune tolerance (reviewed in [1]). However, there are some controversies [37]. As LSECs rapidly dedifferentiate in culture [39, 40] and cells are cultured for several days in lymphocyte stimulation experiments, the in vivo contribution of LSECs in adaptive immunity may be difficult to extrapolate from in vitro experiments. There may also be species differences. We therefore investigated the basal expression of gene products associated with antigen processing and presentation (GO:0019882), and lymphocyte co-stimulation (GO:0031294) in rat LSECs and KCs (Additional file 7). The expression of tap-transporters, immunoproteases, and lysosomal enzymes involved in processing and intracellular traffic of antigens, were similar in the transcriptomes and proteomes of both cells except for Ctse (cathepsin E) and Ctss (cathepsin S) which were significantly more abundant in the KCs (Fig. 8a-b). Expression of MHC class II genes was detected in both cells, but significantly higher in KCs (Fig. 8c). Concerning co-stimulatory molecules, LSECs expressed significantly higher levels of some gene products involved in activation of T-cells (Cav-1, Dpp4, Cd40, Cd320, and Efnb1), while KCs showed an abundance of gene products from the B7/CD28 superfamily (Cd80, Cd86, Btla, Icoslg) (Fig. 8d). Btla, Icoslg, and Cd4 were expressed in both cells, but significantly higher in KCs. BTLA [64], and CD4 [65] are also reported in human LSECs.

### A minor subset of rat LSECs expresses the pan leukocyte marker CD45

**Fig. 9:**
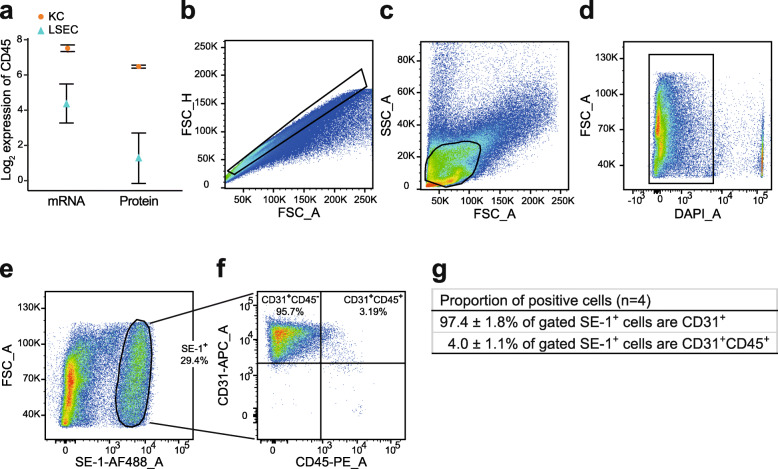
Expression of the leukocyte marker PTPRC/CD45 in rat LSECs. **a**: Ptprc/Cd45 mRNA and PTPRC/CD45 protein expression as obtained from RNA-seq (log_2_ RPKM) and label-free proteomics (log_2_ iBAQ) in KCs (red dots) and LSECs (blue triangles). **b-f**: Representative sequential gating during flow cytometry analysis of rat non-parenchymal liver cells (*n* = 4). Cells were labeled with antibodies to SE-1/FcγRIIb2, CD31, and CD45 (listed in Table 1). **b**: Gating based on the FSC-A vs FSC-H profile to exclude duplets and aggregates from the subsequent analysis. **c**: Gating on the SSC-A vs FSC-A profile to select small cells with limited complexity (enriched in endothelial cells). **d**: Gating to select DAPI negative live cells. **e-f**: LSECs were then identified as SE-1-Alexa488^+^ cells (**e**), and the biexponential CD45-PE/CD31-APC of events (**f**) were used to display and select the CD45^+^ CD31^+^ subsets of LSECs. FMOs (used for gating), and single antibody staining controls are shown in Additional file 8. **g**: Average of the percentage of viable parent populations observed in 4 biological replicates (±standard deviation, SD)

CD45 is reported to be widely expressed in rat LSECs, with high expression in periportally located LSECs, and low expression in mid-zonal LSECs [38, 66]. We here report low expression of CD45 in the LSEC transcriptome, and an even lower expression in the LSEC proteome compared to KCs (Fig. 9a). In order to explore this further, we did flow cytometry of NPCs, and CD45 and stabilin-2 double immune labelling of rat liver sections (Additional file 8). We did not observe a clear co-localization of CD45 with the LSEC marker stabilin-2 in the sinusoids, suggesting either absence or low expression of CD45 in rat LSECs in general or expression in a small subpopulation of these cells. We then performed multicolor flow cytometry (Fig. 9b-f) of rat liver NPCs labeled with antibodies to CD45, SE-1/FcγRIIb2 (specific LSEC marker), and CD31 (pan endothelial cell marker; Additional file 8). NPCs from the 25–45% Percoll gradient interface were used instead of SE-1-MACS-isolated LSECs to eliminate any selection bias. Using strict gating (Additional file 8), we found that 4.0% (±1.06, *n* = 4) of small, low complex, live-gated SE-1^+^ cells were CD31^+^CD45^+^ (Fig. 9g), suggesting expression of CD45 in a small subpopulation of LSECs.

LSECs from normal liver have been reported to not express CD31on the cell surface [67] but in our flow cytometry experiments (Fig. 9g) this marker was shown to be expressed in 97.4% (±1.80, n = 4) of SE-1 positive cells. Immune staining of liver sections showed positive staining in all vasculature, albeit weaker in LSECs than in other endothelia (Additional file 8).

## Discussion

The liver cells facing the blood are represented almost entirely by KCs and LSECs. These two cells make up the most important clearance system for removal of blood borne macromolecules and particles that are incompatible with blood homeostasis [5]. This avid scavenger activity thus fulfills a central role in liver immunity [1, 2] but at the same time poses a serious challenge, namely unwanted uptake of large molecule drug compounds [23]. Curiously, few studies have been undertaken to determine similarities and differences between LSECs and KCs on gene expression and/or proteome levels. Only two comprehensive studies, both done in the inbred C57Bl/6 mouse, have compared liver resident cell populations at the proteome level [41, 42]. The study by Azimifar et al. [41] focused on the distinct functional roles of various hepatic cell types in cholesterol flux, cellular trafficking, and growth receptor signaling, whereas Ding et al. [42] presented an integrated omics analysis focusing on communication and co-ordination between hepatocytes and NPCs, in particular KCs. Against this background we found it timely to carry out a high-throughput mRNA transcriptome and proteome expression study of the two types of specialised hepatic scavenger cells in rat, and focus on the analysis of immune function genes. We chose the outbred Sprague Dawley rat to cover a wide number of genotypes. This rat strain has been widely used in LSEC blood clearance and hepatotoxicity studies [16].

Our omics analysis revealed expression of a great number of genes related to immune functions in both cells. As expected in non-stimulated cells, most of these genes were expressed at low density; however, the great number of expressed immune genes supports the central role for both cells in liver immunity. LSECs seem to be unique among endothelial cells in this respect. Nolan et al. [35] used microarray profiling to compare primary microvascular endothelial cells isolated from liver and several other organs in C57BL/6 mice, and found significant heterogeneity between transcriptomes of the different endothelial cell populations. We did DAVID enrichment analysis [54, 55] on the liver specific gene list (Additional file 9) obtained by pairwise comparison of their LSEC gene expression data (GEO public database-Series GSE47067 [35]) with expression data for other organ-specific endothelial cells included in their study, and found enrichment of terms associated with immune functions in the LSECs (Additional file 9). Neither this mouse study [35], nor our present study in rat address the possibility of the existence of functionally different LSEC subpopulations. A recent single-cell transcriptomics analysis of human liver cells grouped the LSECs into two populations, of which the group enriched in LSECs from the acinar midzone and central venous zone displayed highly enriched immune pathways [68]. This supports the existence of functionally distinct LSEC subpopulations.

Reliable omics studies of isolated cell populations require access to highly pure cell preparations. Several popular markers used to identify LSECs have been associated with controversies regarding their sensitivity, specificity, selection bias, or lack proper validation [36, 37]. Here, we used SE-1-based MACS [43] to purify rat LSECs. This method utilizes the specific targeting of FcγRIIb2 [45], and has been previously reported to yield highly pure LSEC preparations [43]. The SE-1-MACS isolated cells in our experiments consisted of > 97% cells displaying the highly characteristic fenestration which is the structural hallmark of LSECs [16]. Moreover, the cell yield was relatively high (30–40 million LSECs per liver), and immune staining of liver sections using this antibody showed continuous staining along all sinusoids, similar to the LSEC specific endocytosis receptor stabilin-2, further validating SE-1/FcγRIIb2 as a reliable LSEC marker in rat. The same co-distribution of SE-1 and stabilin-2 in rat liver was reported by [46]. Moreover, flow cytometry of rat NPCs showed that 97.4% of SE-1/FcγRIIb2 positive cells were also CD31 positive, supporting their endothelial identity. Rat LSEC expression of CD31 was confirmed by positive staining in rat liver sections, albeit more weakly than in endothelial cells in other vessels, consistent with [69]. Of note, CD31 is upregulated in LSECs in liver inflammation [36, 69, 70]. CD31 has been reported in KCs. However, a recent study employing macrophage and endothelial reporter mice concluded that what seemed to be a population of CD31 positive KCs after FACS were instead contaminating endothelial cells [71]. CD31 is regularly used as endothelial marker in studies of KC functions in mice [72]. In the present study CD31 staining was only observed along vessel structures in the liver tissue, and co-localized with stabilin-2 in the sinusoids.

The expression level and intralobular distribution of FcγRIIb2, and other LSEC markers may vary between species. Recently, the lack of periportal expression of FcγRIIb, and LYVE-1, another commonly used LSEC marker, was reported on immune stained human liver sections [58], suggesting that isolating LSECs from human liver using these receptors as targets may introduce selection bias [36]. Notably, we found that CD36, a recommended LSEC marker in human liver [58], showed low gene and protein expression in rat LSECs, and high expression in KCs, consistent with a previous report in Sprague Dawley rat showing positive immune labeling for CD36 in KCs, but not in LSECs [59]. This shows a clear difference in the cellular distribution of CD36 in rat and human liver.

Interestingly, liver inflammation and fibrosis further affect the LSEC molecular phenotype, leading to downregulation of LYVE-1 in liver cancer and cirrhosis [73], and of FcγRIIb in non-alcoholic steatohepatitis [74]. These studies show that the optimal choice of markers of LSECs and KCs depends on animal species and the health condition of the liver.

Lack of consensus markers and heterogeneity in KCs pose challenges for rat KC isolation. As rat KCs universally express complement receptors for inactivated complement component 3b [75], we used anti-rat-CD11b/c to isolate KCs by MACS with good cell yields. Staining of liver sections showed a scattered distribution with the majority of positive cells located in the periportal region where most KCs reside [76]. However, selection bias towards subpopulations of KCs cannot be excluded, as we found that CD68 positive cells showed a wider distribution within the hepatic lobule than CD11b/c positive cells. Nonetheless, both markers showed the highest density of positive cells in the periportal region. Compared to the extensive literature in mice on the origin of KCs and differences in cell marker expression in subpopulations of liver macrophages [77], little is known about rat liver macrophage subpopulations and markers. In mouse, liver resident macrophages are reported to have the CD11b low, or CD11b negative phenotype, and the CD11hi phenotype includes bone marrow macrophages that have migrated to the liver [78]. The CD11b/c MACS-purified liver macrophages in our study expressed high levels of CRIg (VSig4), CD68 and CD163, which are validated markers of resident KCs [79–81], indicating that they are KCs, but our study cannot confirm whether some have been recruited from bone marrow. However, the cells were isolated from young, healthy rats with normal livers, and the livers were perfused free of blood with perfusion buffer before starting the recirculation system with collagenase buffer in order to disperse the cells, which minimizes the risk of isolating blood monocytes. The source of macrophages in liver has been reported to affect expression levels of enzymes and receptors [82]. Interestingly, two recent studies in mice showed that bone marrow-derived resident liver macrophages, and KCs of yolk sac origin have highly similar gene expression profiles, that is different from that of monocytes [72, 83].

CD45 is used as a negative selection criterion for isolation of human and mouse LSECs [36], whereas the same marker has been reported to be expressed in rat LSECs [38, 84]. In our study we observed a low expression of this marker in the LSEC transcriptomes and proteome compared to KCs, and flow cytometry showed that 4% of SE-1 positive small NPCs with limited complexity were CD31^+^CD45^+^ cells, which indicates CD45 expression in a small subpopulation of rat LSECs. Expression of CD45 in rat LSECs has been linked to recruitment of LSECs from bone marrow [66, 84].

Several SRs and C-type lectins were expressed at high density in the rat LSECs and KCs. This enables the two cells to recognize a wide variety of foreign and endogenous, modified substances, thus maintaining homeostasis [5]. In LSECs, the very high expression of stabilin-1, stabilin-2, FcγRIIb2, and the macrophage mannose receptor suggests that these are the crucial receptors contributing to the remarkably high endocytic capacity of LSECs [5, 16, 18]. In contrast to stabilin-1/-2 and the FcγRIIb2 which are LSEC specific in liver, the mannose receptor is also abundant in KCs. This receptor has traditionally been associated with M2 polarized macrophages but is present in LSECs of all mammalian species examined (rat, mouse, pig, human), while its expression in KCs varies between studies [5]. Interestingly, the macrophage mannose receptor has been reported to be absent in human KCs [85]. Functional studies in rat show that after intravenous injection of soluble ligands for this receptor, such as lysosomal enzymes [86–88], C-terminal procollagen propeptides [13], or ovalbumin [89], much of the ligand is rapidly cleared from blood by uptake in LSECs, which show a higher uptake per cell than in KCs. This points to LSECs as more efficient pinocytic cells.

We also observed mRNA expression in LSEC of some SRs not previously reported in these cells at steady state - including members of SR-class A (Marco), SR-class D (Cd68, Ackr3), SR-class G (Lgals3bp, Cxcl16) and SR-class I (Cd5l, Cd163 and Dmbt1). However, these SRs were either not detected or showed low expression in the LSEC proteome. This may be due to post-transcriptional, translational, and/or protein degradation regulation, and/or the effect of the isolation procedure which may affect the expression. A previous microarray study [39] indicated changes in rat LSEC gene expression already after 2 h in culture. Importantly, proteomics is biased towards identification of highly abundant proteins. Despite high LSEC and KC purity in our experiments we cannot exclude that a low level in the other cell type might result from minute numbers of contaminating cells for some of the genes. Nonetheless, the expression pattern of these SRs was similar to that of LRP1, which has been functionally validated in LSECs [90], and CD45, which we showed by flow cytometry to be expressed in 4% of the LSECs. This suggests that a minor subset of LSECs may express these markers. However, this needs to be further explored in single cell experiments in rat liver cells.

Immune regulatory factors are important in maintaining liver homeostasis, and their dysregulation causes sustained inflammation. In the present study of cells at steady state we found that LSECs predominantly expressed Csf1, Ccl24 and Cxcl12, which affect recruiting, maintenance and homeostasis of other immune cells [63, 91, 92]. Our observation that LSECs express the chemokine scavenger receptor Ackr3 suggests a role for these cells in creating chemokine gradients and thus regulating the overall immune milieu of the sinusoids. KCs on the other hand, more abundantly expressed cytokines such as Il1b and TNF-α known to affect LSEC endocytic functions [93]. In accordance with a proteomics study in mice [42], rat KCs were also more tuned to positive regulation of complement activation by higher expression of triggers of complement activation. Interestingly, rat LSECs and KCs respond to inflammatory mediators in a generally similar manner, developing into pro- and anti-inflammatory subpopulations, indicating that both cells contribute to innate immune responses in liver [94].

Furthermore, we found that rat LSECs express gene products associated with processing and presentation of antigen required for activation of naïve (CD4^+^ and CD8^+^) T cells. Most of these genes were significantly lower expressed in LSECs than in KCs and their function in rat LSECs will need further validation. This finding nevertheless supports functional studies in mouse models concluding that LSECs are antigen presenting cells [1, 32, 95–98]. In physiological conditions, LSECs contribute to generation of T regulatory cells and induction of immune tolerance. However, after fibrotic liver injury due to hepatotoxins, mouse LSECs become proinflammatory, and induce an immunogenic T cell phenotype [99]**.**

## Conclusions

Good resolution was achieved between rat LSECs and KCs, enabling reliable and comprehensive molecular characterization of the cells at steady state. The study showed complementarity of scavenging and immune functions in LSECs and KCs. Both cells expressed high levels of SRs and immune lectins, of which some were present in both cells. Of note, inter-species expression differences for some receptors, as evident from the literature, highlight the need for thorough studies on gene expression in different animal models. We propose that the many common phenotypic and functional traits shared between LSECs and KCs is a consequence of the specialized sinusoidal environment along with the functional demand of the sinusoid, causing the cells to develop complementary and overlapping functions. Our study underlines the importance of taking both cells into consideration in studies of liver immunity. Furthermore, LSECs and KCs play a major role in the, often unwanted, liver uptake of large molecule biopharmaceuticals and nano-formulations [23] preventing drugs from reaching their intended targets. Of note, major off-target drug accumulation in these cells may cause LSEC toxicity, which subsequently may result in liver toxicity. Our results contribute to understanding these uptake mechanisms to a greater detail, which is a prerequisite to develop remedies to reduce unwanted liver uptake.

## Methods

### Animals and ethics statement

Sprague Dawley, Crl:CD (SD), male rats, aged 6–11 weeks were used in the experiments. The animals were obtained directly from Charles River Laboratories (Sulzfeld, Germany). The rats were group housed (3 rats per cage) in 1354G Eurostandard type III conventional cages (Tecniplast, Italy) with aspen bedding (Scanbur, Norway), and with nesting material, houses, and aspen bricks (all from Datasand Ltd., Manchester, UK) as environmental enrichment. The rats were housed under controlled conditions (21 °C ± 1 °C, relative humidity 55% ± 10%, and 12 h light/12 h dark cycle) at the specific pathogen free animal research facility at the University of Tromsø (UiT) – The Arctic University of Norway. The rats had free access to water and standard chow (RM1-E, Special Diet Service, UK), and were acclimatized for at least one week before experiments. Prior to the experiment and during acclimation period, animal health was assessed daily by experienced animal technicians. The experimental protocols and animal handling were approved by the competent institutional authority and the National Animal Research Authority at the Norwegian Food Safety Authority (Mattilsynet; Approval IDs: 4001, 8455, and 0817), and experiments were performed in compliance with the European Convention for the protection of Vertebrate Animals used for Experimental and Other Scientific Purposes. A total of 25 rats were used in this study. All animals were euthanized. While in deep surgical anesthesia (for anesthesia protocol see Method section “Rat liver perfusion, LSEC and KC isolation, and cell purity evaluation”), the vena cava was cut causing exsanguination. For liver tissue sampling for immune histochemistry, the animal was euthanized by CO_2_ according to the requirement in Directive 2010/63/EU in a pre-set system ensuring gradual fill and appropriate exposure time (“Automatic CO_2_ Delivery System”, Vet Tech Ltd., UK), and organs were sampled from the dead animal.

### Rat liver perfusion, LSEC and KC isolation, and cell purity evaluation

Non-parenchymal liver cells (NPCs) were isolated essentially as described in [100], with some modifications. The surgical procedure was performed in the morning (between 8 a.m. and 10 a.m.) in the animal research facility at UiT - The Arctic University of Norway. The rats (body weight 200-320 g) were anesthetized with either 1) a combination of ketamine hydrochloride (Ketalar 50 mg/mL; Pfizer, Norway) and medetomidine hydrochloride (Domitor vet 1 mg/mL, Orion Corporation, Finland); dose of mixture: 0.15 mL Ketalar/100 g BW and 0.05 mL Domitor /100 g BW, administered subcutaneously; or 2) with a mixture (ZRF-mix) of zolazepam /tiletamine hydrochloride 12.9/12.9 mg/mL (Zoletil forte vet, Virbac, Norway), xylazine 1.8 mg/mL (Rompun, Bayer Nordic, Norway) and fentanyl 10.3 μg/mL (Actavis, Norway); dose of mixture: 2 mL/kg BW, administered intraperitoneally. Anesthetic depth was assessed prior to and during the operation procedure to ensure deep surgical anesthesia. The abdomen was opened in the midline, and the intestines gently pushed to the side in order to expose the liver and portal vein. A catheter connected to a peristaltic pump driven perfusion system was inserted into the portal vein and fixed to the vein by a suture, and the caudal vena cava was cut to allow outflow of buffer from the liver and exsanguination of the animal. The liver was then separated from the surrounding tissues by cutting all ligaments and placed on a mesh on the top of a cylinder, where run-through buffer was collected. The liver lobes were perfused free of blood with 250 ml of a calcium-free HEPES-based buffer [100], then perfused for 10 min (flow rate 30 ml/min) in a recirculation system, with 50 ml of a calcium-containing HEPES-based buffer [100] with 0.6 mg/ml collagenase (Worthington, Lot: X4B7108, Worthington Biochemical Corp., Lakewood, NJ). Hepatocytes were sedimented by low speed differential centrifugation (50 g, 2 minx3) leaving mainly NPCs in the supernatant which was decanted and centrifuged (300 g, 10 min). The resulting pellet was resuspended, loaded onto a two-step Percoll gradient (GE Healthcare, Uppsala, Sweden), and centrifuged at 1350 g for 30 min. Cells at the 25–45% Percoll interface, enriched in KCs and LSECs, were collected. To purify LSECs, NPCs were incubated with the M-rSE-1 antibody targeting FcγRIIb2 (CD32b) [45] (Table 1) for 30 min at 4 °C in autoMACS rinsing solution with 1% BSA (Miltenyi Biotec Norden AB, Lund, Sweden), washed, and incubated with anti-mouse IgG2a + b MicroBeads for 30 min at 4 °C. To purify KCs, NPCs were incubated with a biotinylated-CD11b/c antibody (Table 1) followed by incubation with Streptavidin MicroBeads. Labeled NPCs were eluted through an LS-column in a MidiMACS Separator (Miltenyi) according to the manufacturer’s protocol. Typical cell yields were 30–40 million LSECs, and 10 million KCs per rat liver. LSECs and KCs were harvested from separate animals to maximize cell yields.

LSECs (0.25 million cells/cm^2^) were seeded in 100 mm tissue culture dishes (RNA-seq: Nunclon, ThermoFisher Scientific, Waltham, MA; Proteomics: Sarstedt, Nümbrecht, Germany) coated with 2.9 μg/ml bovine collagen type I (Advanced BioMatrix, San Diego, CA, Cat.#5005), in RPMI-1640 cell culture medium supplemented with 20 mM sodium bicarbonate, 0.0006% penicillin, and 0.01% streptomycin (Sigma-Aldrich, St. Louis, MO, Cat.#R8758), and allowed to attach for 1 h. KCs (0.17 million cells/cm^2^) were seeded on uncoated 100 mm dishes, and incubated for 30 min. The cells were then gently washed with pre-warmed (37 °C) medium before extraction of RNA for high throughput RNA-sequencing, or protein for non-label quantitative proteomics.

The purity and morphology of MACS-isolated cells were assessed by phase contrast microscopy (all cultures), scanning electron microscopy (SEM; LSECs for transcriptomic and proteomic analyses, and KCs for proteomic analyses), and immune cytochemistry (LSECs and KCs for proteomic analyses) using antibodies against glial fibrillary acidic protein (GFAP; stellate cell marker), stabilin-2 (LSEC marker) [24, 25], CD11b/c, and SE-1/ FcγRIIb2 (Table 1). The cells for purity assessment by SEM and immune cytochemistry were from the same preparations and cultured in parallel to the cells used for omics experiments, and were seeded in similar density, incubated and washed as for the omics experiments.

### LSEC and KC mRNA transcriptome sequencing

Total RNA was extracted with the RNeasy Mini Kit (Qiagen, Hilden, Germany, Cat.#74,104). PolyA-enriched RNA from LSECs was then purified by MicroPoly(A)Purist™ Kit (Life Technologies, ThermoFisher Scientific), whereas Dynabeads® mRNA DIRECT™ Micro Kit (Ambion, ThermoFisher, Cat.#61,021) was used to purify mRNA from KCs. Quality and quantity of mRNA were measured with Agilent RNA 6000 Pico Kit (Agilent Technologies, Santa Clara, CA, Cat.#5067–1513). The mRNAs were fragmented and reverse transcribed by Ion Total RNA-Seq Kit v2 (Life Technologies) according the manufacturer’s instructions. Three LSEC transcriptome libraries representing 3 biological replicates, each from one individual rat, and three KC transcriptome libraries (3 biological replicates; each from the pooled KC mRNA from 2 rats) were constructed. Templates were prepared by Ion OneTouch™ 200 Template Kit v2 DL and Ion PGM™ Sequencing 300 Kit, loaded on Ion 316 chips, and sequenced with the Ion Torrent Personal Genome Machine (Life Technologies). We generated 1.09 billion nucleotide sequence data from the LSEC pool, corresponding to approximately 8.2 million mapped reads, and 0.93 billion nucleotide sequence data from the KS pool, corresponding to 6.5 million mapped reads. Additional file 10 lists the information on number of raw reads, reads after trimming, average length of the trimmed sequences and the number of reads mapped to the reference genome for each biological replicate.

### Transcriptomic data analyses

Bioinformatics analyses were performed with the CLC Genomics Workbench 8.0.2 (Qiagen® Bioinformatics), and the Bioconductor project. Raw sequencing reads were subjected to adaptor trimming, followed by quality trimming (Ambiguous limit = 2 and Quality limit = 0.05). Based on quality reports the reads were filtered based on length (minimum 15 and maximum 300 nucleotides); then 10 nucleotides from the 5′ end, and 20 nucleotides from the 3′ end were removed. All samples from the 6 experiments (LSECs, *n* = 3; KCs, n = 3) were included in the analysis as they were deemed homogenous with respect to 5-mer analysis and GC contents, and were free of ambiguous bases. RNA-seq analysis was performed with CLC Genomics Grid Worker 7.0.1. The reads were mapped to *Rattus norvegicus* reference genome (Rnor_6.0 [101], which generated the gene expression counts and RPKM (reads per kilobase of exon model per million mapped reads [56]) values. Other parameter values used in mapping were: mismatch cost = 2, insertion cost = 3, deletion cost = 3, length and similarity fraction = 0.8 each, allowed maximum number of hits for a read = 10, and map to inter-genic regions. We used the edgeR (3.28.0)-limma (3.42.0) workflow as described in [102] to analyze the gene-level count data, using the following criteria: genes with low expression were filtered out using the filterByExpr function, and the remaining genes were considered to be expressed and were used in subsequent data analyses. Heteroscedasticity of the data was removed with voomWithQualityWeights function available in the limma package [103], after trimmed mean of M-values (TMM) normalization.

### Preparation of samples for quantitative proteomics, and tandem mass spectrometry (LC-MS/MS)

MACS-isolated cells were allowed to adhere for 30 min (KC, *n* = 4 biological replicates, each from one individual rat) or 1 h (LSECs, n = 3 biological replicates, each from one individual rat) to 100 mm petri dishes as described under “Rat liver perfusion, LSEC and KC isolation, and cell purity evaluation”. The cells were washed with RPMI-1640 (37 °C) to remove non-adherent cells, then immediately scraped out in triethylammonium bicarbonate (TEAB) solution (ThermoFisher) to collect protein lysate, which was centrifuged to remove cellular debris. Protein pellets were resuspended in 2 M urea and 50 mM TEAB. Samples of 20 μg protein were digested for 6 h in 1:100 (w/w) Lysyl Endopeptidase® (Fujifilm Wako Chemicals Europe GmBH, Neuss, Germany), then diluted to 1 M urea and digested overnight with 1/20 (w/w) trypsin (V511A, Promega Corporation, Madison, WI). OMIX C18 tips (Varian Inc., Palo Alto, CA) were used for sample cleanup and concentration. Peptide mixtures containing 0.1% formic acid were loaded onto the Thermo Fisher Scientific EASY-nLC1000 system and EASY-Spray column (C18, 2 μm, 100 Å, 50 μm, 50 cm). Peptides were fractionated using a 2–100% acetonitrile gradient in 0.1% formic acid over 50 min at a flow rate of 250 nl/min. Separated peptides were analyzed using Thermo Scientific Q-Exactive mass spectrometer. Data was collected in data dependent mode using a Top10 method.

### Label-free proteomics analyses

Raw files from the Q-Exactive MS/MS were analysed using the quantitative proteomics software MaxQuant [104] (version 1.5.6.0). Proteins were identified using the built in Andromeda search engine using the UniProtKB *Rattus norvegicus* database (Jan 2017). Main search peptide tolerance was set to 4.5 ppm and MS/MS mass tolerance was set to 20 ppm. An FDR ratio of 0.01 was needed to give a protein identification. At least 2 peptides had to be quantified to give a quantitation value.

To estimate protein abundance, iBAQ values (i.e. the sum of peak intensities of all tryptic peptides matching to a specific protein divided by the number of theoretically observable peptides [105]) were generated with MaxQuant, and used for downstream quantitative proteomic analysis with Perseus (version 1.6.02). Perseus, R statistical computing (version 3.4.1), and Bioconductor (version 3.5) environments were used for bioinformatics and statistical analyses. The generated list of proteins was filtered to remove protein hits that were annotated as only identified by site, contaminants and reverse hits in Perseus. All samples for proteomics were run twice on LC-MS/MS and the median of the iBAQ values of the two runs was considered as the expressed iBAQ value. The annotation of the protein IDs and the corresponding genes were carefully curated. The iBAQ values of all protein IDs corresponding to a specific gene were added to remove redundancy in gene annotation. The resulting iBAQ values were then scaled to make an equal column sum. Protein with low expression were filtered using the filterByExpr function in edgeR-limma. The filtered data were rescaled to per million using the cpm function, followed by TMM normalization. The term “iBAQ” in figures and text refers to these normalized values and were used in the subsequent analyses. The same edge-R-limma workflow as used in the RNA-seq data analysis was used for the subsequent differential analysis of the proteomics data.

### Data integration and visualization

In order to compare RNA-seq data with proteomics data the expression of gene products in the RNA-seq dataset that corresponded to protein IDs in the proteomics data were reevaluated by summing up the counts of all relevant genes. Log_2_ transformed expression values with prior addition of an offset of 1 were used in the visualization, unless mentioned otherwise.

### Immune labeling of cells and liver tissue

MACS isolated LSECs and KCs (parallel cultures to proteomics experiments) were seeded on collagen coated glass coverslips (LSECs) or uncoated glass coverslips (KCs) at similar density as with the omics experiments, and incubated and washed in the same way, before fixation 15 min in 4% paraformaldehyde (PFA) in PBS, pH 7.2. NPCs from the 25–45% interface of the Percoll gradient were seeded on collagen-coated glass coverslips, incubated for 1 h, washed and fixed 15 min in 4% PFA. Liver samples were embedded in TissueTek OCT compound (Sakura Finetek, Zoeterwoude, Netherlands), snap frozen in liquid nitrogen, and stored at − 70 °C. Cryosections, 8–10 μm, were fixed in cold acetone for 10 min, then incubated in blocking buffer for 1 h, and immune labeled. All antibodies (Table 1) were diluted in blocking buffer, which was 1% BSA and 2% goat serum in PHEM buffer (w/v: 1.81% PIPES, 0.65% HEPES, 0.38% EGTA, 0.1% MgSO_4_), pH 7, when labeling cryosections, and 1% BSA in tris buffered saline, 0.05% Tween 20, pH 8.4, when labeling cells. Sections and cells were incubated with primary antibody at 4 °C overnight, then washed and labeled with secondary antibody for 1 h at room temperature. Isotype controls or non-immune IgG controls were used in all immune staining experiments. Nuclei were stained with DAPI (1:1000 in PBS; Sigma-Aldrich). Confocal microscopy was performed using a Zeiss LSM780 system (Carl Zeiss, Oberkochen, Germany). For purity assessment by differential counting of immune labeled MACS isolated cells, images were taken from 5 different areas of the cultures, including at least 350 cells from each CD11b/c-MACS isolation, and 700 cells from each SE-1-MACS isolation in the differential cell count.

### Scanning Electron Microscopy (SEM)

SE-1-MACS-isolated LSECs (parallel cultures to proteomics experiments) were seeded on collagen coated 24-well tissue culture plates for 1 h, whereas CD11b/c MACS-isolated KCs were seeded for 30 min on uncoated 24-well plates. Cells were gently washed with medium before fixation in McDowell’s fixative (4% PFA, 1% glutaraldehyde, in phosphate buffer, pH 7.2). Fixed cultures were stamped out from the plate and cells processed for SEM using the following protocol: 1) 3x wash in PHEM buffer, pH 7; 2) 1 h incubation with 1% tannic acid in PHEM; 3) 3x wash in PHEM; 4) 30 min in 1% osmium tetroxide in H_2_O; 5) 3x wash in PHEM; 6) dehydration in graded ethanol (30–100%); 7) drying in hexamethyldisilazane (Sigma-Aldrich). Specimens coated with 10 nm gold/palladium were scanned and imaged in a Zeiss Sigma Field Emission Scanning electron microscope (Carl Zeiss) at 2 kV. For cell purity assessment, high resolution overview images were taken at random from at least 5 different areas per cell culture (LSEC and KC samples for proteomics experiments), or at least 3 areas per culture (LSEC samples for RNA-seq experiments). Cells from all areas were included in the differential cell count, including at least 600 cells per KC sample, and 800 cells per LSEC sample in the cell purity assessment for proteomics, and 280–470 cells per LSEC sample in the cell purity assessment for RNA-seq.

### Flow cytometry

Samples of 0.5-1 × 10^6^ NPCs collected from the 25–45% interface of the Percoll gradient were stained with antibodies to CD45, CD31, and SE-1/FcγRIIb2 (Table 1) at 4 °C in dark for 20 min. Data acquisition and analysis were performed in a BD LSRFortessa™ Cell Analyzer (BD Biosciences, San Jose, CA) with BD FACSDiva Software version 8.0.1. The laser configuration and the PTM voltage were calibrated prior running the samples. The PTM voltage was adjusted during the experiments using the single stained controls. The data were further quality checked and analyzed with FlowJo V10.7.1 software (BD Biosciences). The AutoSpill/AutoSpread spillover algorithm available in FlowJo 10.7.1 was used to address the compensation issue using single stained controls post acquisitions. Isotype controls, single antibody controls, and FMO controls were used to properly interpret the acquired data. DAPI staining was performed to discriminate between live and dead NPCs. An excess of 100,000 events were recorded and analyzed in every test within each biological replicate (*n* = 4), each representing one individual rat.

### Statistical tests

The descriptive and inferential statistical analyses and graphical plots of the transcriptomics and proteomics data were performed either in the R/Bioconductor or the Perseus environment. RNA-seq analysis was performed with CLC Genomics Grid Worker 7.0.1. The genes/proteins retained after filtering of low expressed gene/protein using filterByExpr function were deemed to be expressed and were used in subsequent data analyses. Heteroscedasticity of the data was removed with voomWithQualityWeights function available in the limma package [103], after trimmed mean of M-values (TMM) normalization. Differential expression analysis of the transcriptomics and the proteomics data was tested with edgeR (3.28.0)-limma (3.42.0) workflow as described in [102], with FDR multiple correction [103], as described under “Transcriptomic data analyses” and “Label-free proteomics analyses”. The genes/proteins were identified as differentially expressed when the |log_2_ fold change| ≥1 and FDR ≤ 0.05. All samples were included in the omics analyses. LSECs and KCs were compared at functional level using gene set enrichment analysis (GSEA) [49, 50] on gene lists ranked based on expression level with priori defined collection of annotated gene sets from Molecular Signatures Database. The gene sets were considered significantly enriched if FDR q-value ≤0.05.

## Supplementary Information

**Additional file 1.** Gene set enrichment analysis. The Excel file (.xls) shows the output of gene set enrichment analysis (GSEA) [49, 50] of pre-ranked gene lists from the rat LSEC and KC RNA-seq datasets, associated with Gene Ontology (GO) biological processes (BP). The genes were pre-ranked based on expression. We have used the C5 collection of annotated gene sets in the Molecular Signatures Database (release 6.2; BP) [53] which consists of gene sets derived from GO [51, 52]. Name of worksheets: “GSEA_plot”, “GSEA_RNAseq_LSEC_BP”, and “GSEA_RNAseq_KC_BP”. The worksheet named “GSEA_Plot” contains the selected enriched BPs shown in Fig. 4.

**Additional file 2.** List of all expressed genes in the RNA-seq and proteomics datasets. Excel file (.xls) with all genes and proteins that were deemed expressed, as defined in Methods, and used in the downstream analysis and visualization of data. The worksheet named “RNA_seq_whole expressed” contains the data from the RNA-seq experiments (expression values in RPKM), and differential expression analysis results. The worksheet named “Proteomics_whole expressed” contains the data from the label-free proteomic experiments (expression values in iBAQ), and differential expression analysis results.

**Additional file 3.** Genes and proteins associated with immune system processes. Excel file (.xls) with the list of genes and proteins associated with the term immune system processes (GO:0002376) presented in the two heatmaps in Fig. 5a, along with their associated expression values (RPKM, or iBAQ). The two worksheets are named “Immune genes_RPKM_RNAseq”, and “Immune genes_iBAQ_proteomics”. Of note, for visualization, log_2_(RPKM+ 1) and log_2_(iBAQ+ 1) were used in the heatmaps in Fig. 5a.

**Additional file 4.** Scavenger receptors and C-type lectins. Excel file (.xls) with the list of genes and proteins presented in the two heatmaps in Fig. 6a, along with their associated expression values (RPKM, or iBAQ). The two worksheets are named “SRs&lections_RPKM_RNAseq) and “SRs&lectins_iBAQ_proteomics”. For visualization, log_2_(RPKM+ 1) and log_2_(iBAQ+ 1) were used in Fig. 6.

**Additional file 5.** Immune histochemistry for CD68. Immune histochemistry of acetone-fixed frozen sections of rat liver showing the distribution pattern of CD68 in the liver lobule. Sections were labeled with an antibody to CD68 (red fluorescence) and stabilin-2 (Stab2, green fluorescence) and subjected to confocal laser scanning microscopy. Antibodies are listed in Table 1. Nuclei were stained with DAPI (blue).

**Additional file 6.** Immune regulatory factors. Excel file (.xls) with the list of expressed genes annotated to cytokine receptor binding (GO:0005126), cytokine receptor activity (GO:0004896), complement activation (GO:0006956), or complement receptor activity (GO:0004875) in rat LSECs and KCs. The file shows their corresponding abundance in the RNA-seq datasets (RPKM values; worksheet named “Immunereg.factors_RPKM_RNAseq”) and label-free proteomics datasets (iBAQ values; worksheet named “Immunereg.factors_iBAQ_LFP”) along with differential expression analysis outputs. For visualization of the selected genes shown in Fig. 7, log_2_(RPKM+ 1) was used.

**Additional file 7.** Genes annotated to antigen processing and presentation and lymphocyte co-stimulation. Excel file (.xls) with the list of expressed genes associated with antigen processing and presentation (GO:0019882), and lymphocyte co-stimulation (GO:0031294) in LSECs and KCs. The file shows their corresponding abundance in the RNA-seq datasets (RPKM values; worksheet named “Immuneactivation_RPKM_RNAseq”) and label-free proteomics datasets (iBAQ values; worksheet named “Immuneactivation_iBAQ_LFP”) along with differential expression analysis outputs. For visualization of the selected genes and proteins shown in Fig. 8, log_2_ (RPKM+ 1), and log_2_ (iBAQ+ 1) were used.

**Additional file 8.** Immune histochemistry for CD31 and CD45, and controls for SE-1, CD31, CD45 flow cytometry experiments. a-b: Immune histochemistry of acetone-fixed frozen sections of rat liver showing the distribution pattern of stabilin-2, CD31 and CD45 in the liver lobule. a: Sections were labeled with antibodies to CD31 (red fluorescence) and stabilin-2 (Stab2, green fluorescence) and subjected to confocal laser scanning microscopy. CD31 stained all hepatic endothelia; in the sinusoids the CD31 staining overlapped with the stabilin-2 staining (arrows). b: Sections labeled with antibodies to CD45 (red fluorescence) and stabilin-2 (Stab2, green fluorescence). a-b: Pv, portal vein/venule. Antibodies are listed in Table 1. Nuclei were stained with DAPI (blue). c: The figure panel contains the contour profiles (of the singlet, small, low complexity, live-gated non-parenchymal liver cells) of the three single antibody staining controls on the different fluorophore channels used during the acquisition of the data in the flow cytometry experiment presented in Fig. 9. d: The figure contains the contour profiles of the three FMO controls and tests used to verify the gating used to interpret the experiment in Fig. 9.

**Additional file 9. **Analysis of microarray expression data obtained from Nolan et al. 2013 [35]. Excel file (.xls) with the comparative analysis of expression data obtained from a microarray profiling study of mouse (*Mus musculus*) primary microvascular endothelial cells, published by Nolan DJ, Ginsberg M, Israely E, Palikuqi B, Poulos MG, James D, et al. Molecular signatures of tissue-specific microvascular endothelial cell heterogeneity in organ maintenance and regeneration [35]. The microarray data was downloaded from the GEO public database-Series GSE47067. Title of dataset: In vivo endothelial cell heterogeneity. The first worksheet, named “Pairwise_DGE (Nolan_2013)”, shows pairwise analysis of the expression data of endothelial cells from different organs. The second worksheet, named “DAVID_ liver specific genes”, presents the DAVID enrichment analysis [54, 55] output of genes that were consistently significantly abundant in mouse liver sinusoidal endothelial cells (LSECs) in every pairwise comparison of LSECs with other microvascular endothelial cells in the dataset (Significance level: FDR ≤ 0.05, and log_2_ fold change ≥1).

**Additional file 10.** Ion Torrent sequencing results. Excel file (.xls) summarizing the Ion Torrent PGM sequencing results, including number of mapped sequences and average lengths.

## Data Availability

The datasets supporting the conclusions of this article are included within the article and its additional files. Additional data generated and analyzed during this study are available from the corresponding author on reasonable request. The RNA-sequencing datasets generated and analysed during the current study are available in the NCBI Sequence Read Archive (SRA). SRA accession number to dataset: PRJNA574898. [https://www.ncbi.nlm.nih.gov/bioproject/PRJNA574898/]. The mass spectrometry proteomics datasets generated and analysed during the current study are available in the ProteomeXchange Consortium PRIDE database. ProteomeXchange accession: PXD012080. [https://www.ebi.ac.uk/pride/archive/projects/PXD012080].

## Abbreviations

FDR: False discovery rate
GO: Gene ontology
GSEA: Gene set enrichment analysis
MACS: Magnetic-activated cell separation
KC: Kupffer cell
LSEC: Liver sinusoidal endothelial cell
NPC: Non-parenchymal liver cell
SR: Scavenger receptor
