# Supplementary material for: Transcriptome and proteome profiling reveal complementary scavenger and immune features of rat liver sinusoidal endothelial cells and liver macrophages
Source: BMC Mol Cell Biol. 2020 Nov 27;21:85. doi: 10.1186/s12860-020-00331-9 (PMC7694354; doi:10.1186/s12860-020-00331-9)
Supplement: Supplementary file 5 — Additional file 5. Immune histochemistry for CD68. Immune histochemistry of acetone-fixed frozen sections of rat liver showing the distribution pattern of CD68 in the liver lobule. Sections were labeled with an antibody to CD68 (red fluorescence) and stabilin-2 (Stab2, green fluorescence) and subjected to confocal laser scanning microscopy. Antibodies are listed in Table 1. Nuclei were stained with DAPI (blue). [file 12860_2020_331_MOESM5_ESM.pdf]

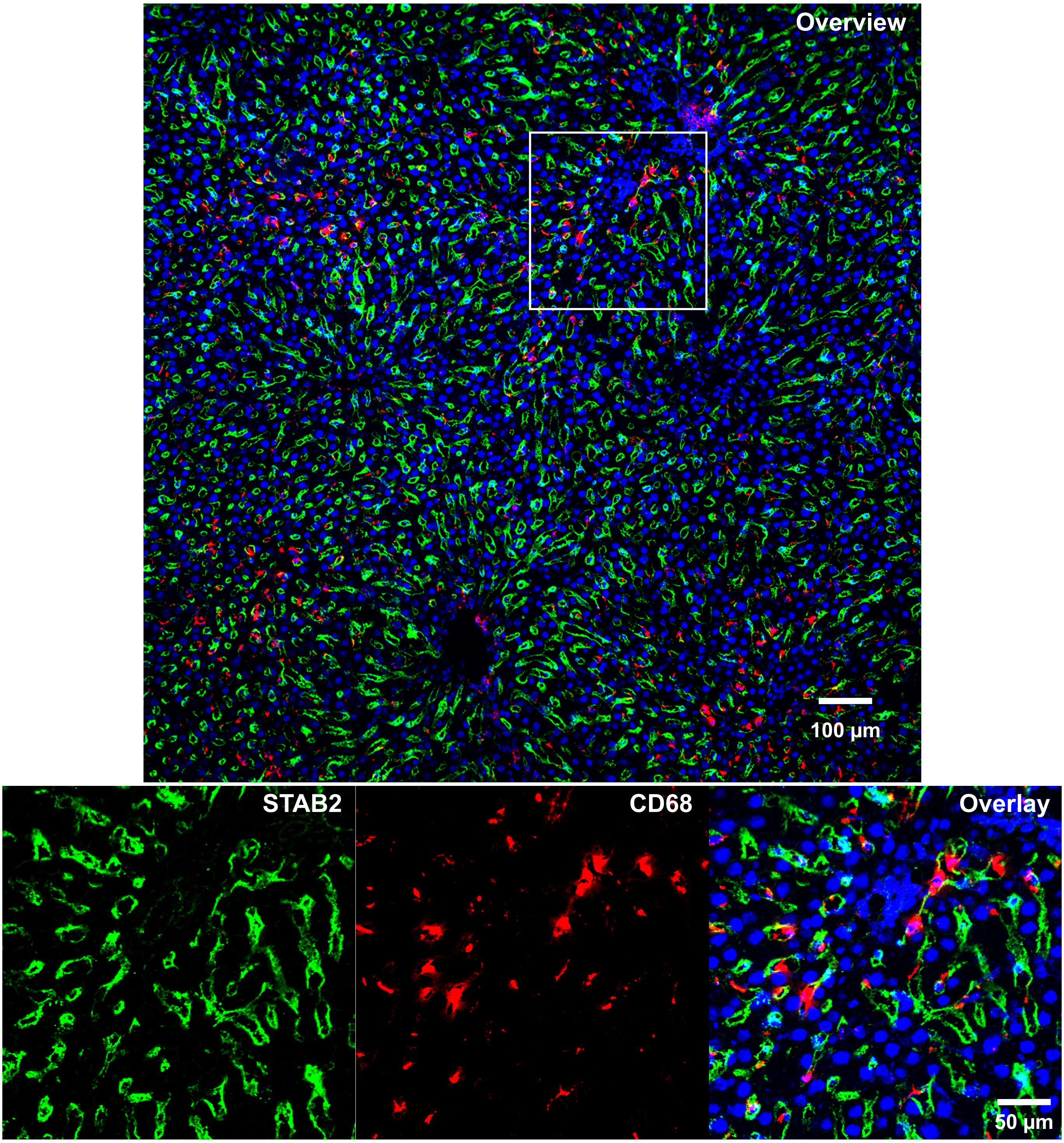

Additional file 5: Immune histochemistry for CD68

Immune histochemistry of acetone-fixed frozen sections of rat liver showing the distribution pattern of CD68 in the liver lobule. Sections were labeled with an antibody to CD68 (red fluorescence) and stabilin-2 (Stab2, green fluorescence) and subjected to confocal laser scanning microscopy. Antibodies are listed in Table 1. Nuclei were stained with DAPI (blue).
