# Supplementary material for: Transcriptome and proteome profiling reveal complementary scavenger and immune features of rat liver sinusoidal endothelial cells and liver macrophages
Source: BMC Mol Cell Biol. 2020 Nov 27;21:85. doi: 10.1186/s12860-020-00331-9 (PMC7694354; doi:10.1186/s12860-020-00331-9)
Supplement: Supplementary file 8 — Additional file 8. Immune histochemistry for CD31 and CD45, and controls for SE-1, CD31, CD45 flow cytometry experiments. a-b: Immune histochemistry of acetone-fixed frozen sections of rat liver showing the distribution pattern of stabilin-2, CD31 and CD45 in the liver lobule. a: Sections were labeled with antibodies to CD31 (red fluorescence) and stabilin-2 (Stab2, green fluorescence) and subjected to confocal laser scanning microscopy. CD31 stained all hepatic endothelia; in the sinusoids the CD31 staining overlapped with the stabilin-2 staining (arrows). b: Sections labeled with antibodies to CD45 (red fluorescence) and stabilin-2 (Stab2, green fluorescence). a-b: Pv, portal vein/venule. Antibodies are listed in Table 1. Nuclei were stained with DAPI (blue). c: The figure panel contains the contour profiles (of the singlet, small, low complexity, live-gated non-parenchymal liver cells) of the three single antibody staining controls on the different fluorophore channels used during the acquisition of the data in the flow cytometry experiment presented in Fig. 9. d: The figure contains the contour profiles of the three FMO controls and tests used to verify the gating used to interpret the experiment in Fig. 9. [file 12860_2020_331_MOESM8_ESM.pdf]

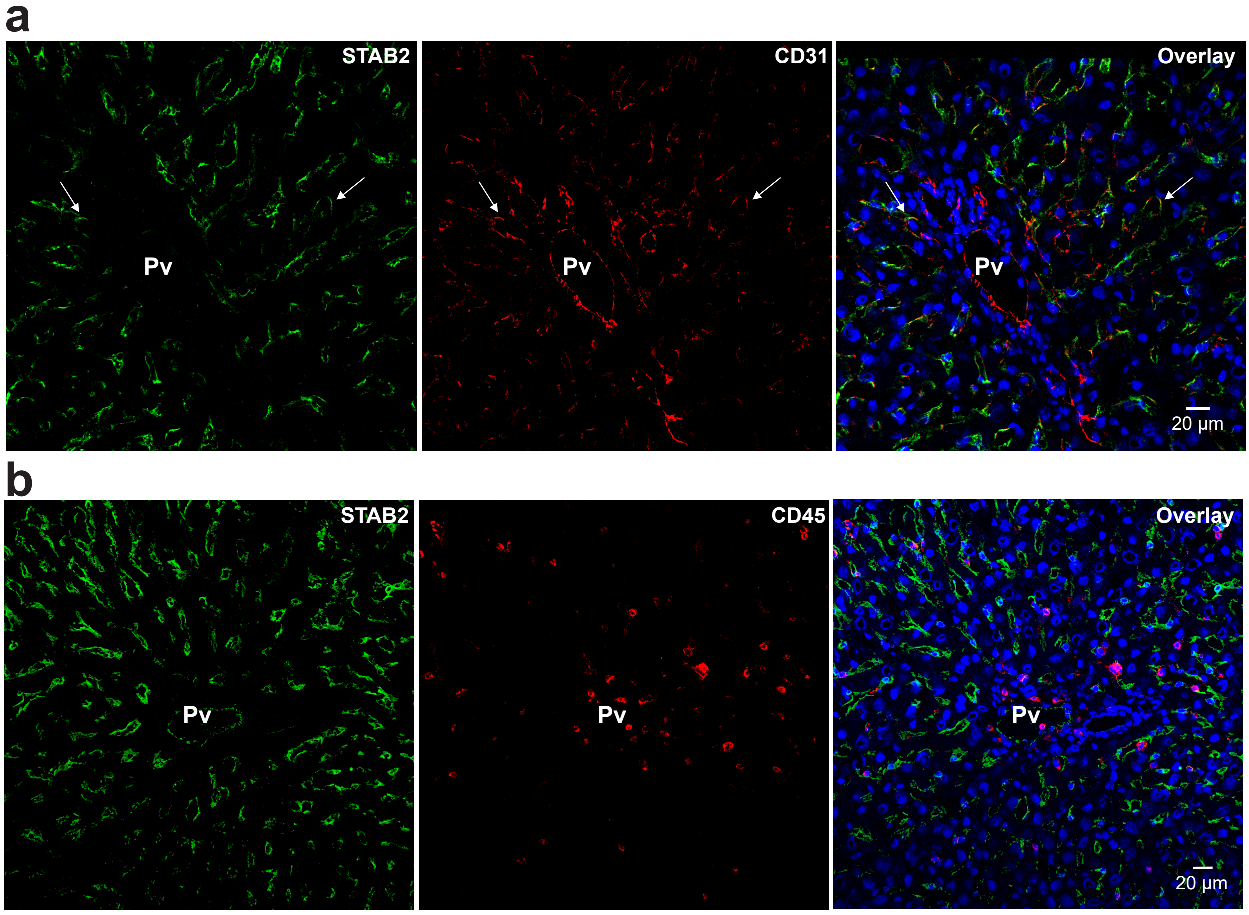
a
STAB2
Pv
CD31
Pv
Overlay
Pv
20 µm
b
STAB2
Pv
CD45
Pv
Overlay
Pv
20 µm

c

Single labeling: CD45

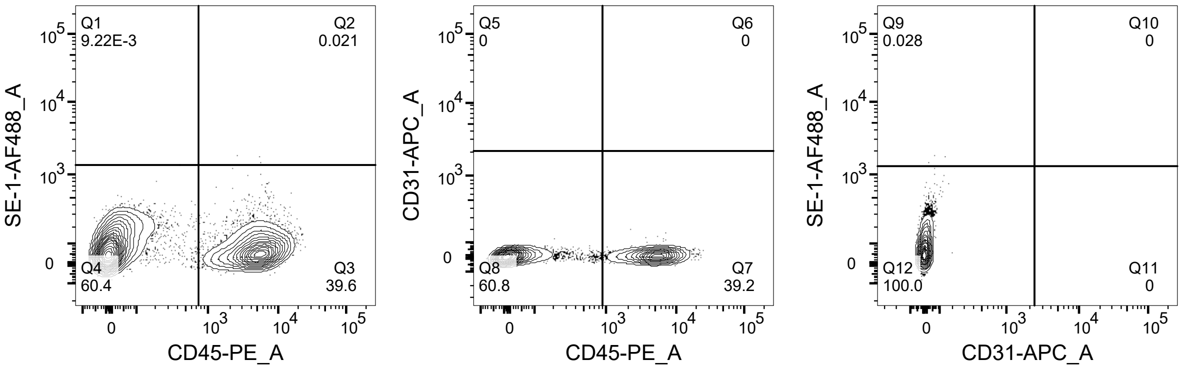

Single labeling: SE-1

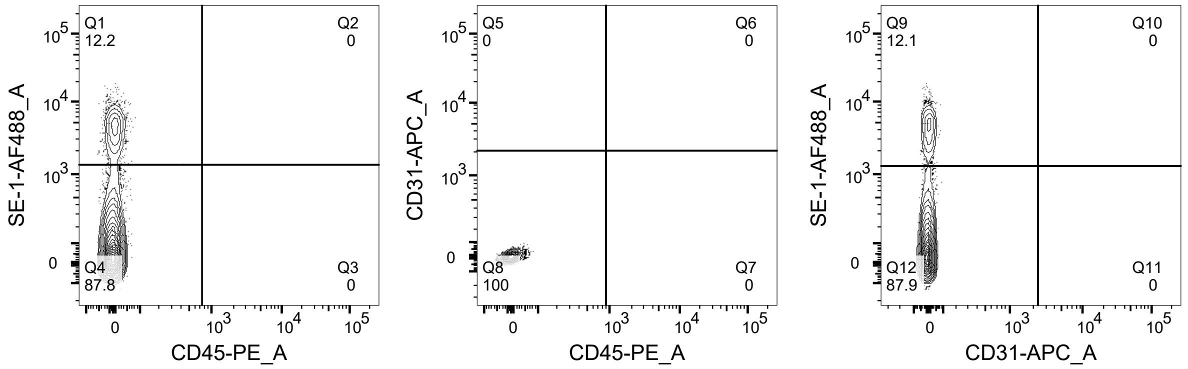

Single labeling: CD31

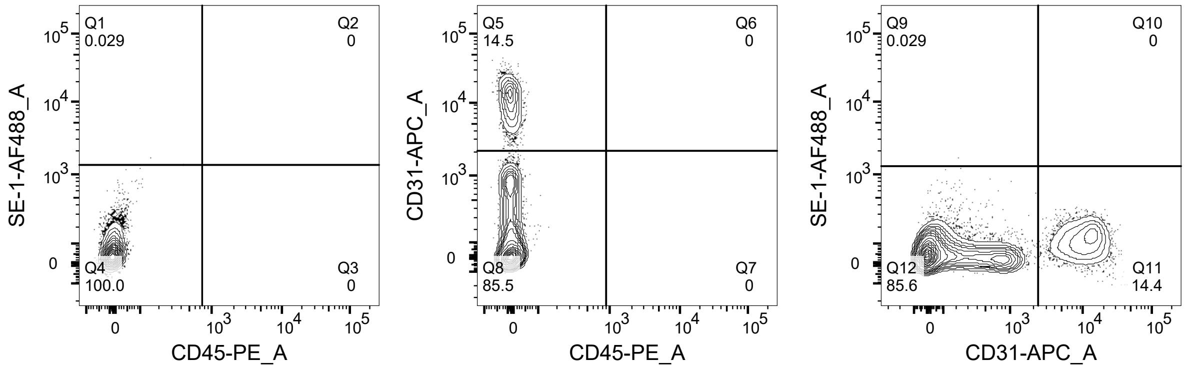

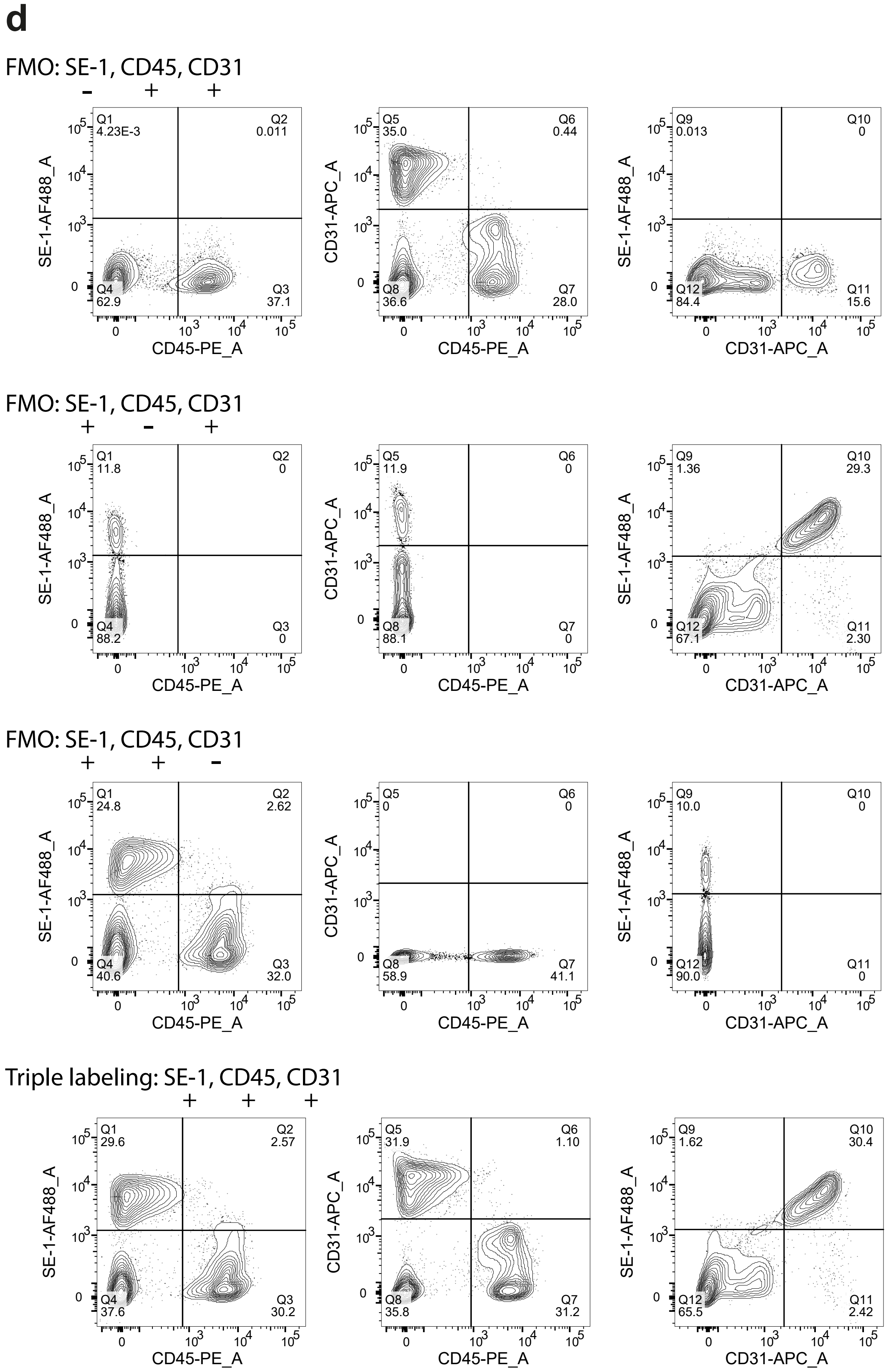

d
FMO: SE-1, CD45, CD31
– + +
Q1 4.23E-3
Q2 0.011
Q4 62.9
Q3 37.1
Q5 35.0
Q6 0.44
Q8 36.6
Q7 28.0
Q9 0.013
Q10 0
Q12 84.4
Q11 15.6
FMO: SE-1, CD45, CD31
+ – +
Q1 11.8
Q2 0
Q4 88.2
Q3 0
Q5 11.9
Q6 0
Q8 88.1
Q7 0
Q9 1.36
Q10 29.3
Q12 67.1
Q11 2.30
FMO: SE-1, CD45, CD31
+ + –
Q1 24.8
Q2 2.62
Q4 40.6
Q3 32.0
Q5 0
Q6 0
Q8 58.9
Q7 41.1
Q9 10.0
Q10 0
Q12 90.0
Q11 0
Triple labeling: SE-1, CD45, CD31
+ + +
Q1 29.6
Q2 2.57
Q4 37.6
Q3 30.2
Q5 31.9
Q6 1.10
Q8 35.8
Q7 31.2
Q9 1.62
Q10 30.4
Q12 65.5
Q11 2.42
SE-1-AF488_A
CD31-APC_A
CD45-PE_A

Additional file 8: Immune histochemistry for CD31 and CD45, and controls for SE-1, CD31, CD45 flow cytometry experiments

a-b: Immune histochemistry of acetone-fixed frozen sections of rat liver showing the distribution pattern of stabilin-2, CD31 and CD45 in the liver lobule. a: Sections were labeled with antibodies to CD31 (red fluorescence) and stabilin-2 (Stab2, green fluorescence) and subjected to confocal laser scanning microscopy. CD31 stained all hepatic endothelia; in the sinusoids the CD31 staining overlapped with the stabilin-2 staining (arrows). b: Sections labeled with antibodies to CD45 (red fluorescence) and stabilin-2 (Stab2, green fluorescence). a-b: Pv, portal vein/venule. Antibodies are listed in Table 1. Nuclei were stained with DAPI (blue).

c: The figure panel contains the contour profiles (of the singlet, small, low complexity, live-gated non-parenchymal liver cells) of the three single antibody staining controls on the different fluorophore channels used during the acquisition of the data in the flow cytometry experiment presented in Figure 9.

d: The figure contains the contour profiles of the three FMO controls and tests used to verify the gating used to interpret the experiment in Figure 9.
